# Abstracts of the 3rd UNICA Sport Science International Conference 2024

**DOI:** 10.1186/s40798-025-00877-y

**Published:** 2025-07-28

## A1 Genetics and epigenetics (GE): Influence of ACTN-3 C.1729C > T (RS1815739) Polymorphism on Match-Play Maximal Running Speed in Elite Football Players: A Preliminary Report

### Allegra G.*^[1]^, Flore L.^[2]^, Secci J.^[1]^, Baldus M.^[1]^, Cugia P.^[1]^, Piras F.^[1]^, Scorcu M.^[1]^, Calò C.M.^[2]^, Tocco F.^[3]^*Massidda M.*^*[3,4]*^

#### ^[1]^ Cagliari Calcio SpA ~ Cagliari ~ Italy ^[2]^Department of Life and Environmental Sciences, University of Cagliari ~ Cagliari ~ Italy, ^[3]^ Department of Medical Science and Public Health, University of Cagliari ~ Cagliari ~ Italy [4] Gdansk University of Physical Education and Sport - Gdansk - Poland.

##### Correspondence:  Massidda M.

*Sports Medicine – Open* 2025, ***11(1)***: A1

**Background** A frequent genetic variant in the alpha actinin-3 gene (ACTN-3, c.1729C > T (rs1815739)) has been linked to the progression of soccer career [2], the prevalence and severity of muscle injuries [3], and elite athletic performance [1]. While the TT genotype appears to have a detrimental impact on football career advancement and the likelihood of developing muscle injuries, the C allele and the CC genotype of the ACTN-3 polymorphism appear to favour the capacity to produce strong and forceful muscle contractions, which are necessary during sprinting performance. A crucial component of contemporary football is high-intensity running, which can distinguish between players at the highest level of competition and those at a lower level [4].

**Objective/Aim** This study examined the influence of a common genetic variant in the alpha actinin-3 gene (ACTN-3, c.1729C > T (rs1815739) on maximal running speed during official matches in elite football players. We hypothesized that football players harbouring the C allele would have a higher maximal running speed (MRS) during matches than those footballers with the TT genotype.

**Methods** MRS was collected, using a Global Position System (GPS) at high sampling frequencies (50 Hz), from 45 elite football players of a same professional football team during 26 official matches for a total of 707 matches observations. Genomic DNA was extracted using a buccal swab, and the ACTN-3 genotype was performed using a RFLP PCR method.

**Results** The main finding was that CC players showed significantly higher MRS than TT players (CC = 33.1 ± 1.3 km h^-1^ vs TT = 31.5 ± 1.9 km h^-1^, * p*= 0.041). Moreover, the players harbouring a copy of the C allele showed a trend toward higher MRS than players carriers of the TT genotype (CC + CT = 32.9 ± 1.5 km h^-1^ TT = 31.5 ± 1.9 km h^-1^* p* = 0.06). The MRS (36.4 km h^-1^) was registered for a player carrier of CC genotype, while the lower running speed (29.8 km h^-1^) was reordered for a player carrier of TT genotype.

**Conclusion and References** For the first time, we discovered an association between elite football players' match-play maximal sprinting speed and the ACTN-3 polymorphism. If our findings will be confirmed in more extensive studies, they may be applied in the future to modify training regimens in order to improve football performance.

**References**

1. Yang et al. Am J Hum Genet. 2003, 73:627–31.
2. Coelho et al. Int J Sports Med. 2018, 39(14):1088–93.
3. Massidda et al. Clin J Sport Med. 2019, 29(1):57–61.
4. Carling et al. J Sport Sci. (2012) 30(4):325–36.

## A2 Genetics and epigenetics (GE): Advances in Genentics of Musculoskeletal Soft Tissue Injuries

### Collins M.*

#### Health through Physical Activity, Lifestyle and Sport Research Centre (HPALS), Division of Physiological Sciences, University of Cape Town (UCT) ~ Cape Town ~ South Africa

##### Correspondence: Collins M.

*Sports Medicine – Open* 2025, ***11(1)***: A2

**Background** Musculoskeletal soft tissue (MSK) injuries are common. While the biological mechanisms of these injuries are complex and poorly understood, they are caused by training load interacting, together with other factors, with an individual's genetic background. A heritability of 40% has been estimated for tennis elbow and as high as 69% for anterior cruciate ligament ruptures. Therefore, it is believed that genetic variants play an important role in predisposing individuals to MSK injuries and contribute to the inter-individual variation in susceptibility to injury.

**Objective/Aim** Advances in the identification of genetic factors associated with MSK injuries will be reviewed.

**Methods** Our present understanding of the contribution of genetic variants in modulating the risk of tendon and ligament injuries will be reviewed. The current and future application of high throughput technologies in identifying associated variants, as well as the role of epigenetic mechanisms in the aetiology of injury and possible clinical applications will be explored.

**Results** To date, most of the research investigating the association of genetic variants with MSK injuries has been hypothesis-driven, where the association of candidate genes, which have been identified based on their biological function in tendons and ligaments, with risk of injuries tested in case–control studies. Genes that encode for structural proteins, such as collagens and proteoglycans, as well as proteins involved in development, homeostasis and remodelling of tendons and ligaments were selected and prioritised. Generally, studies have explored candidate genes in single populations or ancestries and when repeated in other cohorts produce conflicting results. Regions of the genome with potential risk-modulating effects in other protein coding, non-protein coding, regulatory and intronic regions have been overlooked using this approach. A limited number of studies have therefore utilised a hypothesis-free approach, whereby the whole genome or exons are investigated to determine all genetic or exonic variants that are associated with MSK injuries. International collaborations have enabled investigators to start testing the association of variants identified by both hypothesis-driven and -free approaches in larger cohorts. Preliminary functional theories have been presented for some of the associated genes. These theories will assist in explaining the molecular mechanisms underlying the injury.

**Conclusion and References** It is critical that genetic loci are identified using hypothesis-free approaches and confirmed in large clinically well well-defined cohorts, as well as in multiple populations. The functional significances of associated variants should be explored at the cellular and tissue levels to enable us to start determining the clinical relevance of these genetic contributions to normal adaptation and injury susceptibility, recovery, and tissue capacity. Finally, epigenetic mechanism also needs to be considered and included into the current injury modules.

## A3 Genetics and epigenetics (GE): Gender-Specific Influence of ACTN3 R577X Polymorphism on Muscle Injury in Professional Brazilian Soccer Players

### De Almeida K.Y.*^[1]^, Guimarães R.D.S.^[2]^, Rocha M.R.^[3]^, Saito M.^[1]^, Kikuchi N.^[1]^

#### ^[1]^Nippon Sport Science University ~ Tokyo ~ Japan, ^[2]^University of Extremadura ~ Extremadura ~ Spain, ^[3]^Federal University of Minas Gerais ~ Minas Gerais ~ Brazil

##### Correspondence: De Almeida K.Y.

*Sports Medicine – Open* 2025, ***11(1)***: A3

**Background** Muscle injuries pose significant challenges for athletes, impacting performance and participation in sports, particularly in soccer where the risk is notably high (Ekstrand, Hagglund, & Walden, 2011). Genetic factors, including ACTN3 R577X polymorphism, are associated with athletic ability and injury susceptibility (Hall et al., 2022). However, while studies have predominantly focused on male athletes, gender-specific differences in injury rates and genetic influences remain underexplored (Sentsomedi & Puckree, 2016).

**Objective/Aim** This study aimed to analyze the incidence and severity of muscle injuries in male and female high-level professional Brazilian soccer athletes throughout one season, considering the impact of the ACTN3 R577X polymorphism and gender differences.

**Methods** The study included 31 males (29.4 ± 4.8 years old, 177.8 ± 8.4 cm, 76.8 ± 9.3 kg) and 28 females (22.7 ± 5.6 years old, 164 ± 0.05 cm, 57.2 ± 6.6 kg) professional soccer athletes from the First Division of the Brazilian Championship. Injury data from the medical records of the soccer club were assessed, and genotype analysis of the ACTN3 R577X polymorphism with saliva samples. Comparisons across groups, were performed using the χ2 test, ANCOVA adjusted by age and Student's t-test.

**Results** A total of 94 non-contact muscle injuries were recorded for the entire cohort during the 2023 season. Female athletes exhibited a trend of higher incidence of muscle injuries per player compared to males (2.6 injuries/athlete in female and 1.8 in male,* p* = 0.06), as well as a trend of more injuries per player compared to males (9.2 ± 7 and 7.6 ± 17, respectively, * p* = 0.05). The presence of the ACTN3 X allele was associated with longer medical leave in the entire cohort (* p* = 0.007), and particularly in females compared to males (* p* = 0.01), and compared to females with the RR genotype (* p* = 0.03).

**Conclusion and References** These results suggest an association between the X allele and the incidence and severity of injuries, with a higher effect of ACTN3 R577X polymorphism in women compared to men. This association can be due to its lower average levels of testosterone, making other parameters, such as the influence of ACTN3, more significative in the development of muscle injuries in females. The findings highlight the importance of considering both genetic factors and gender-specific differences in muscle injury research.

**References**

1. Ekstrand, J., Hagglund, M., & Walden, M. (2011). Injury incidence and injury patterns in professional football: the UEFA injury study. Br J Sports Med, 45(7), 553–558.
2. McCall, A., Carling, C., Nedelec, M., Davison, M., Le Gall, F., Berthoin, S., & Dupont, G. (2014). Risk factors, testing and preventative strategies for non-contact injuries in professional football: current perceptions and practices of 44 teams from various premier leagues. Br J Sports Med, 48(18), 1352–1357.
3. Sentsomedi, K. R., & Puckree, T. (2016). Epidemiology of injuries in female high school soccer players. Afr Health Sci, 16(1), 298–305.

## A4 Genetics and epigenetics (GE): Novel MYBPC3 Genetic Variants Associated with Polish Elite Athletes Status

### Dzitkowska-Zabielska M.*, Bojarczuk A., Cieszczyk P.

#### Gdansk University of Physical Education and Sport ~ Gdansk ~ Poland

##### Correspondence: Dzitkowska-Zabielska M.

*Sports Medicine – Open* 2025, ***11(1)***: A4

**Background** The adaptation of an athlete's body to competitive sports is conditioned not only by hard training but also by genetic predispositions. A large and growing body of literature has investigated the impact of many polymorphic variants on changes in athletic performance, and conflicting results have been obtained. In competitive sports, the ability of the heart muscle to remodel as a result of intense exercise is critical. The MYBPC3 gene encodes for the thick-filament associated protein cardiac myosin-binding protein C, thus contributing to the maintenance of sarcomeric structure and regulation of contraction and relaxation. A meta-analysis of GWAS among athletes identified rs1052373 (TT homozygotes) in the MYBPC3 gene to be associated with endurance athlete status. Athletes who were TT homozygotes had a higher VO2max than those who carried the CC and CT alleles.

**Objective/Aim** This study is aiming to examined polymorphic variants in the MYBPC3 gene and their association with high level athletes' status.

**Methods** 101 male elite Polish athletes and 41 healthy individuals from the Polish population were analyzed. Data was extracted from whole-genome sequencing (WGS), using the following parameters: paired reads of 150 bps, at least 90 Gb of data per sample with 300 M reads, and 30 × mean coverage.

**Results** rs3729953, rs3729948, rs58912703, rs10769255, rs11570051 in the MYBPC3 gene have been identified as significant in the athlete's group. All of them are being reported for the first time.

**Conclusion and References** MYBPC3 may play an important role in the heart adaptation to intense exercise by modulating cardiac contraction during intense exercise, which causes heart remodeling to compensate for elevated blood pressure by increasing systolic volume (producing a large cardiac output). To confirm these data, further analyses, using larger group should be conducted.

**References**

1. The Relationship between Changes in MYBPC3 Single-Nucleotide Polymorphism-Associated Metabolites and Elite Athletes' Adaptive Cardiac Function.
2. Riguene E, Theodoridou M, Barrak L, Elrayess MA, Nomikos M. J Cardiovasc Dev Dis. 2023 Sep 18;10(9):400. 10.3390/jcdd10090400.
3. Genes and Athletic Performance: The 2023 Update. Semenova EA, Hall ECR, Ahmetov II. Genes (Basel). 2023 Jun 8;14(6):1235. 10.3390/genes14061235.
4. Advances in sports genomics. Ahmetov II, Hall ECR, Semenova EA, Pranckevičienė E, Ginevičienė V. Adv Clin Chem. 2022;107:215–263. 10.1016/bs.acc.2021.07.004.

## A5 Genetics and Epigenetics (GE): Do Genetic Variation Influence Motor Performance in Young Soccer Players?

### Flore L.*^[1]^, Massidda M.^[2]^, Desogus D.^[3]^, Congiu B.^[1]^, Cherchi M.^[1]^, Scorcu M.^[4]^, Tocco F.^[2]^, Calò C.M.^[1]^

#### ^[1]^Department of Life and Environmental Sciences, University of Cagliari ~ Cagliari ~ Italy, ^[2]^Department of Medical Sciences and Public Health, University of Cagliari ~ Cagliari ~ Italy, ^[3]^Sport and Exercise Sciences Degree Courses, University of Cagliari ~ Cagliari ~ Italy, ^[4]^Cagliari Calcio SpA ~ Cagliari ~ Italy

##### Correspondence: Flore L.

*Sports Medicine – Open* 2025, ***11(1)***: A5

**Background** Human physical performance is influenced by environmental and genetic factors. The physical determinants of elite youth soccer players are specific to the stage of maturity. However, it is unknown if genetic variation can be responsible for different speed and power among elite youth soccer playing at different stages of maturity (1). Most of the previous studies focused on adult soccer players, but the contribution of genetic variation to phenotypes involved in physical performance in young soccer players remains still unknown.

**Objective/Aim** The aim of this research was to verify the genetic influence on motor test ability in young soccer players.

**Methods** DNA was extracted from buccal swab from 66 male young soccer players (10.88–14.37 years old). Written informative consent was obtained for each participant, signed by one of the parents. The following markers, previously correlated with sport performance, have been genotyped:

- rs6265 in the brain-derived neurotropic factor (BDNF) gene;
- rs1815739 in the alfa actinin (ACTN3) gene;
- rs8192678 in Peroxisome proliferator-activated receptor gamma coactivator 1-alpha (PPARGC1A) gene;
- rs4341 in angiotensin-converting enzyme (ACE) gene;
- rs5810761 in Bradykinin receptor B2 (BDKRB2) gene.

The following motor tests were performed by each players: a) Standing Long Jump (SLJ), to verify the lower limb explosive muscle strength, b) Loughborough Soccer Dribbling Test (LSDT), to test the football dribbling ability at high velocity, c) speed test (ST), to evaluate the running speed on 5 m, 10 m, 20 m, and d) Harre Circuit Test (HCT), to assess coordinative abilities. The best value has been considered. Logistic regression analysis, through SNPSTAT program, was applied to evaluate the genotype/phenotype association.

**Results** All the polymorphisms met the Hardy Weinberg equilibrium. The markers showed the following MAF values: ACE*D = 0.28, ACTN3*X = 0.26, BK2*D = 0.41, BDNF*Met = 0.27, PPARG*A = 0.33. The logistic regression analysis reveals no significant association for HCT and SLJ. For LSDT, we found just a borderline result in terms of statistical significance (*p* = 0.05) for BDNF. Indeed, assuming the dominant model individuals with Val/Val genotype showed higher value (14.5) compared to their counterpart (13.9). ST turned out interesting results: for the 5 m sprint we found only a value at the limit of significance (* p* = 0.06) for ACTN3, showing XX individuals slower than the counterpart, in the recessive model (1.4 vs 1.2).

For the sprint at 10 m significant results are shown by ACE (*p* < 0.0001) and PPARG gene (*p*= 0.017), assuming both recessive models, precisely, individual with II and GG genotypes turned out to be faster. Sprint at 20 m confirms the association with ACTN3 in the recessive model (XX significantly slower, *p* = 0.047).

**Conclusion and References** Our research suggests that genetic factors can influence motor ability already at a young age, suggesting a great genetic basis for speed and motor control.

**References**

1. Murtagh CF, et al. J strength Cond Res. 2018; 32: 297–303.

## A6 Genetics and Epigenetics (GE): Genetic Profile of Elite Sprint and Endurance Athletes

### Gineviciene V.*

#### Faculty of Medicine, Vilnius University ~ Vilnius ~ Lithuania

##### Correspondence: Gineviciene V.

*Sports Medicine – Open* 2025, ***11(1)***: A6

**Background** The interplay of environmental factor, together with genetic profile, is an essential prerequisite for attaining high-level athletic status.

**Objective/Aim** The aim of the present research was to investigate the association of 26 DNA polymorphisms (previously identified as markers for sprint/power and endurance phenotypes) with elite athlete status in Lithuania.

**Methods** A total of 250 Lithuanian athletes (112 endurance-oriented, 138 sprint/power-oriented) and 250 healthy unrelated individuals (controls) were genotyped (PCR–RFLP or Real-Time PCR) for 26 gene polymorphisms (AGT rs699; AGTR1 rs5186; AGTR2 rs11091046; ACE rs4646994; MB rs7293; ACTN3 rs1815739; AMPD1 rs17602729; CKM rs8111989; NOS3 rs2070746; MSTN rs11333578, MSTN rs1805086; GALNT13 rs10196189; CREM rs1531550; MCT1 rs1049434; MPRIP rs6502557; GPC5 rs852918; PPARGC1A rs8192678; PPARGC1B rs7732671; PPARA rs4253778; PPARG rs1801282; PPARD rs2016520; TFAM rs1937; HIF1A rs11549465; VEGF rs2010963; Il6 rs1800795; BDNF rs6265) which most were previously reported to be associated with athlete status or related intermediate phenotypes. Vertical jump (counter-movement) test was used for the estimation of the short-term explosive muscle power. Anaerobic alactic maximum power was estimated by a stair climbing test. Aerobic capacity function was determined using maximum oxygen consumption (VO₂max). Anthropometric measures were also estimated.

**Results** The results showed that the phenotypic indices of the Lithuanian athletes correspond to the elite levels. The genotypes of the investigated polymorphisms were different with respect to gender and associated with phenotypic indices. The athletes, carriers of the ACTN3 TT, CKM AA, ACE DD, NOS3 TT, PPARGC1A AA, PPARA CC, AMPD1 CC, BDNF CT, HIF1A CT, MB GG, AGT TT, AGTR1 CC, GPC5 GG MPRIP AA genotypes are associated with sprint/power performance and have better ability to achieve high muscle efficiency in short-term, maximum-effort requiring physical activity. The athletes, carriers of MSTN (delA), ACTN3 CT, AMPD1 CC, GDF5 GG, AGTR1 AA, ACE DD, MB AA, TFAM CC, PPARGC1A GG, PPARA GG, PPARG CC and CG genotypes, typically have better aerobic capacity (and VO₂max value).

**Conclusion and References** We conclude that the studied polymorphisms can be regarded as markers associated with predisposition to sprint/power or endurance athletics. Significance and relevance of this research is supported by the fact that understanding of molecular processes through athlete’s genetic analysis is essential to the creation and implementation of personalized training programs for athletes in future.

## A7 Genetics and Epigenetics (GE): Genetic Variants and Personality Traits in Athletes

### Huminska-Lisowska K.*, Michalowska-Sawczyn M., Cieszczyk P., Grzywacz A.

#### Gdansk University of Physical Education and Sport ~ Gdansk ~ Poland

##### Correspondence: Huminska-Lisowska K.

*Sports Medicine – Open* 2025, ***11(1)***: A7

**Background** Athletic success is influenced not only by physical ability, but also by psychological resilience and personality traits. These traits are shaped by a complex interplay of genetic and environmental factors, not just by training and experience. Recent advances in psychogenetics have begun to reveal how specific genetic polymorphisms and epigenetic modifications influence personality traits, providing insights into the biological underpinnings that may predict athletic performance.

**Objective/Aim** Our research explores the impact of genetic and epigenetic variations on athletes' adaptation to physical stress and personality traits. By examining the associations between genetic markers, epigenetic changes, and personality traits, the study aims to identify key biomarkers that can predict athletic performance. This includes elucidating how these genetic factors contribute to psychological endurance and the dynamics of performance in sports.

**Methods** This case–control study included elite endurance, strength, and martial arts athletes, as well as non-athletic controls. Psychometric assessments were performed using the NEO Five-Factor Inventory (NEO-FFI) and the Temperament and Character Inventory (TCI-R) questionnaire. Biological samples were collected for DNA isolation, genotyping by real-time PCR, and epigenetic profiling by DNA methylation analysis. Statistical analyses were performed using STATISTICA 13 software.

**Results** Our research revealed significant associations between specific polymorphisms and key personality traits. By analyzing polymorphisms in genes such as DRD2 (rs1800498) or BDNF (rs6265) and assessing DNA methylation profiles, significant associations were found between specific genetic markers and key personality dimensions such as conscientiousness or extraversion. In addition, the COMT (rs4680) and HTR1A (rs6295) polymorphisms influenced psychological profiles by enhancing traits such as novelty seeking, self-managenent, and stress management, which are essential for peak performance in f.e. martial arts. These associations highlight the influence of both genetic predisposition and exercise-induced epigenetic modifications on psychological resilience and performance in athletes.

**Conclusion and References** In summary, this study highlights the significant role of genetic and epigenetic factors in shaping the psychological and physical profiles of athletes. By linking specific genetic polymorphisms and epigenetic profiles to personality traits and resilience, the findings provide a deeper understanding of the biological underpinnings of athletic performance. The identification of these biomarkers not only advances our knowledge of sports science, but also paves the way for personalized training programs that can optimize an athlete's performance based on their unique genetic makeup.

The National Science Center in Poland funded the project (2016/21/B/NZ7/01068).

## A8 Genetics and Epigenetics (GE): The Col11a1 Gene is Associated with Risk of ACL Rupture in a Large International Cohort: A Preliminary Study

### Laguette M.*^[1]^, Alfasi A.^[1]^, Firfirey F.^[1]^, Cieszczyk P.^[2]^, Ficek K.^[3]^, Häger C.^[4]^, Stattin E.^[5]^, Nilsson K.^[6]^, Alvarez-Rumero J.^[7]^, Eynon N.^[7]^, Feller J.^[8]^, Tirosh O.^[9]^, Posthumus M.^[1]^, September A.^[1]^, Collins M.^[1]^

#### ^[1]^University of Cape Town ~ Cape Town ~ South Africa, ^[2]^Department of Physical Education, Gdansk University of Physical Education and Sport. ~ Gdansk ~ Poland, ^[3]^Faculty of Physiotherapy, Jerzy Kukuczka Academy of Physical Education in Katowice ~ Katowice ~ Poland, ^[4]^Department of Community Medicine and Rehabilitation, Umeå University ~ Umeå ~ Sweden, ^[5]^Department of Immunology Genetics and Pathology, Science for Life Laboratory, Uppsala University ~ Uppsala ~ Sweden, ^[6]^Department of Surgical and Perioperative Sciences, Umeå University ~ Umeå ~ Sweden, ^[7]^Institute for Health and Sport (iHeS), Victoria University, Melbourne ~ Victoria ~ Australia, ^[8]^OrthoSport Victoria, Epworth Healthcare, Melbourne ~ Victoria ~ Australia, ^[9]^School of Health Sciences, Swinburne University of Technology, Melbourne ~ Victoria ~ Australia

##### Correspondence: Laguette M.

*Sports Medicine – Open* 2025, ***11(1)***: A8

**Background** A growing body of evidence suggests an association between genetic variants within collagen genes and risk of musculoskeletal soft tissue injuries. ACL rupture is an acute injury with a lengthy recovery time and a high cost. Type XI and type V collagen play a crucial role in fibrillogenesis. Alterations to the properties of tissue structures as a result of genetic variations could predispose individuals to injuries. Variants within type XI genes, together with type V collagen variants, were previously implicated in the risk of Achilles tendinopathy in a combined South African (SA) and Australian (AUS) population.

**Objective/Aim** Investigate the independent association of variants within the type XI collagen genes, namely COL11A1 rs1676486 G > A, COL11A1 rs3753841 A > G, COL11A2 rs1799907 A > T and COL11A2 rs16868943 G > A with ACL rupture risk in a large combined cohort.

**Methods** A large case–control genetic association study was conducted comprised of 1329 physically active and unrelated participants from SA (n = 459), Sweden (SWE n = 211), Poland (POL n = 291), and AUS (n = 368). Genotyping was performed using Taqman© SNP genotyping assays and the QuantStudio 3™ Real-Time PCR System and software (Applied Biosystems, Waltham, MA, USA). Statistical analysis was performed using R statistical packages.

**Results** The COL11A1 rs1676486 G/G genotype was significantly associated with increased risk of ACL ruptures in the combined cohort (* p* = 2.7 X 10–6, OR: 0.57, 95% CI: 0.45—0.72). Similarly, the G/G genotype was also independently associated with increased ACL rupture risk in the POL and SWE sporting population group. No other associations were noted.

**Conclusion and References** The COL11A1 rs1676486 G/G genotype was associated with increased risk of ACL ruptures in the large combined cohort. This work supports the hypothesis that variants within the type XI collagens may be involved in susceptibility to ACL injuries. Interestingly, this variant is associated with risk of other musculoskeletal injuries such as lumbar disc displacement. The next objective would be to examine risk modulation in combination with type V collagen genes as they interact at the molecular level as well as in the context of the risk of Achilles tendinopathy. Importantly, sex specific associations and gene–gene interactions must be explored. Deciphering the molecular basis and altered risk modulation for these genetic associations remains a priority towards a better understanding of the aetiology of these conditions and identifying novel therapeutic targets.

## A9 Genetics and Epigenetics (GE): Polymorphisms of Candidate Genes Associated with the Incidence and Severity of Noncontact Sports Injuries in Spanish Adolescents. LES-5G Study

### Lim T.*^[1]^, Yvert T.^[2]^, Barceló-Guido O.^[3]^, López-Nuevo C.^[3]^, Benady C.^[1]^, Gómez-Cobo N.^[3]^, Pérez-Ruiz M.^[2]^, Iturriaga T.^[3]^, Santiago C.^[1]^

#### ^[1]^Department of Rehabilitation, Faculty of Health Sciences and Sport, Universidad Europea de Madrid ~ Madrid ~ Spain, ^[2]^Department of Health and Human Performance, Faculty of Sciences of Sport and Physical Activity-INEF, Universidad Politécnica de Madrid ~ Madrid ~ Spain, ^[3]^Department of Sport Sciences, Faculty of Health Sciences and Sport, Universidad Europea de Madrid ~ Madrid ~ Spain

##### Correspondence: Lim T.

*Sports Medicine – Open* 2025, ***11(1)***: A9

**Background** 70,6% of Spanish children and adolescent are not complying with the WHO recommendations on the minimum diary levels of moderate and vigorous physical activity(1). According to the literature, one of the main reasons for abandoning sport in children and adolescents is the appearance of injuries(2). Therefore, reducing the risk of injury in this population would reduce sports abandonment, promote participation in sports throughout life, and produce improvements in public health associated with regular sports practice(3). Football is one of the sports with the highest incidence of soft tissue injuries(4), especially in adolescents(5).

During the last decade, interesting research focused on the genetic aspect of sport injuries, and several polymorphisms were found to be related to the risk of noncontact injury(6). However, most of these studies only analyse adult athletes; therefore, there is a lack of knowledge about adolescent athletes.

**Objective/Aim** To determine whether there is an association between candidate genetic polymorphisms and the incidence or severity of injury in Spanish adolescent football players.

**Methods** 74 adolescent football players from youth categories of a first division Spanish team, aged between 12 and 17 years were analysed. The incidence and severity of injuries were collected for the season 2022–2023. The genotypes of polymorphisms ACTN3 rs1815739, COL5A1 rs12722, IL6 rs1800795, GEFT rs11613457, MMP3 rs679620 and DCN rs516115 were analysed using Taqman™ Genotyping Assays.

**Results** Significant differences were found for the polymorphism COL5A1 rs12722: the TT genotype carriers had a higher incidence of injury per 1000 h of practice: 1.94 ± 2.04 vs. 0.768 ± 1.64 for the CC + CT carriers, * p* = .005, Cohen’s d = 0.649 (medium effect). Furthermore, carriers of the TT genotype had a higher risk of severe injury (more than 28 days of absence): 33.3% had severe injuries during the season vs. 8.7% for the other genotypes (CC + CT), * p* = 0.030, Cramer’s V = 0.312 (moderate effect).

Significant differences were found for the DCN rs516115 polymorphism: the TT genotype carriers had a higher incidence of injury per 1000 h of practice: 1.79 ± 2.12 vs. 0.759 ± 1.52 for the CC + CT carriers, * p* = 0.015, Cohen’s d = 0.565 (medium effect). Furthermore, the TT genotype carriers had a higher risk of severe injuries: 26.5% had severe injuries during the season vs. 10.0% for the other genotypes (CC + CT), * p* = 0.002, Cramer’s V = 0.387 (moderate effect).

No significant differences were found for the remaining polymorphisms, nor using genotype scores.

**Conclusion and References** Polymorphisms COL5A1 rs12722 and DCN rs516115 in genes related to the composition of the extracellular muscle matrix present genotypic differences in the incidence and severity of noncontact sport injuries.

**References**

1. PASOS 2022: www.gasolfoundation.org
2. Habelt 2011: PMID:22,355,484
3. Watson 2019: PMID:31,659,001
4. López-Valenciano 2019: PMID:31,171,515
5. Moreno Pascual 2008: 10.1016/S0211-5638(08)72954-7
6. Lim 2021: PMID:34,270,833

## A10 Genetics and Epigenetics (GE): Are DCN Gene Variants Associated with ACL Rupture Susceptibility?

### Losinska K.^[1]^, Ficek K.^[2]^, Huminska-Lisowska K.^[1]^, Leonska-Duniec A.^[1]^, Cieszczyk P.*.^[1]^

#### ^[1]^Faculty of Physical Culture, Gdansk University of Physical Education and Sport ~ Gdańsk ~ Poland, ^[2]^Academy of Sport Education in Katowice ~ Katowice ~ Poland

##### Correspondence: *Cieszczyk P.*

*Sports Medicine – Open* 2025, ***11(1)***: A10

**Background** Anterior cruciate ligament (ACL) injuries present significant challenges in sports medicine, manifesting in various forms from sprains to complete ruptures. Decorin (DCN) is a gene implicated in the structural integrity of ligaments and cartilage, playing a crucial role in connective tissue organization. The role of genetic variations within the DCN gene in the susceptibility and frequency of ACL injuries is a growing area of interest. Understanding these genetic influences suggests the potential for targeted preventive strategies and personalized treatment approaches based on individual genetic predispositions.

**Objective/Aim** This study aims to examine the association between single nucleotide polymorphisms (SNPs) in the DCN gene and the frequency of ACL injuries (ACLF), focusing on distinguishing individuals with a single injury from those with multiple injuries. The study also evaluates the genetic associations with different injury severities, including sprains (ACLS), partial ruptures (ACLRP), and complete ruptures (ACLRC).

**Methods** A case control genetic association study was conducted using DNA samples from healthy participants.

without a history of ACL injuries and surgically diagnosed ACLR participants All samples were genotyped for the DCN: rs516115 and rs13312816 variants.

**Results** Both SNPs conformed to the HWE (rs516115, * P* = 1.0; rs13312816, * P* = 0.605). The single-locus analysis indicated that individuals with the A/G genotype of SNP rs516115 have an increased odds of multiple ACL injuries (OR = 6.03) compared to the A/A genotype, with the G/G genotype further amplifying this risk (OR = 9.71). The dominant model highlighted a stronger genetic predisposition (OR = 6.48) toward recurrent ACL injuries for those carrying A/G and G/G genotypes, while the recessive model revealed a significant increase in risk (OR = 3.88) for individuals with the G/G genotype. Similarly, the A/T genotype of SNP rs13312816 was associated with a higher risk (OR = 3.19) of recurrent ACL injuries. The haplotype-based analysis for ACLF revealed that, in reference to the DCN [A:T] haplotype (frequency 0.7195), the [G:A] haplotype (frequency 0.0852) was associated with an OR of 3.34 (95%CI: 1.33—8.35), and the [G:T] haplotype (frequency 0.1953) with an OR of 4.79 (95%CI: 2.35—9.79). No significant associations were found for ACLS, ACLRP, and ACLRC phenotypes.

**Conclusion and References** Our study underscores the significant association of specific genotypes and haplotypes within the DCN gene with an increased frequency of ACL injuries, particularly highlighting the genetic predisposition for individuals experiencing multiple injuries. The presence of the G allele in both SNP rs516115 and the [G:A] and [G:T] haplotypes are marked as risk factors for recurrent ACL injuries. These findings emphasize the potential of genetic markers in identifying individuals at higher risk for frequent ACL injuries, supporting the development of personalized prevention and rehabilitation strategies.

## A11 Genetics and Epigenetics (GE): Podological Assessment of Lower Limb Injuries in Rugby Players and Potential Implications of Recombinant TP-84 Glycosylase-Depolymerase Destroying Capsules

### Lubkowska B.*^[1]^, Mazurek T.^[2]^, Skowron P.M.^[3]^

#### ^[1]^Faculty of Physical Education, Gdansk University of Physical Education and Sport ~ Gdansk ~ Poland, ^[2]^Department of Orthopaedics and Traumatology ~ Gdansk ~ Poland, ^[3]^Department of Molecular Biotechnology, Faculty of Chemistry, University of Gdansk ~ Gdansk ~ Poland

##### Correspondence: Lubkowska B.

*Sports Medicine – Open* 2025, ***11(1)***: A11

**Background** Athletes, particularly rugby players, frequently encounter lower extremity injuries due to the demanding nature of their sport. These injuries, ranging from fractures to soft tissue damage, often involve the nail apparatus. Recognizing the frequency and characteristics of these injuries is crucial for effective podiatric management. However, traditional antibiotic therapy, while commonly employed for wound infections, presents challenges including microbial resistance and performance hindrance in athletes.

**Objective/Aim** This project aimed to explore alternative strategies for combating infections, particularly those necessitating antibiotic use. Specifically, the focus was on finding novel approaches to treat wound infections common among athletes, given their susceptibility to injuries that breach skin integrity and deeper tissues. Recombinant TP-84 depolymerase, known for its ability to disrupt bacterial envelopes, was investigated for its potential therapeutic application.

**Methods** A comprehensive podiatric evaluation was conducted, including the management of subungual hematomas and ingrown nails in rugby players. Clinical observations and outcomes of antibiotic therapy were recorded to understand injury management. Additionally, advanced experimental work involved molecular cloning of the TP84_26 gene into an Escherichia coli expression vector. Genetic expression of recombinant depolymerase-glycosylase was carried out, along with isolation and purification of the native enzyme [1]. Functional tests assessed the efficacy of the native and recombinant enzyme in degrading bacterial capsules and biofilms from the "Bacillus" group [2].

**Results** Examination of lower limb conditions among rugby players revealed various abnormalities associated with epidermal disruption. Despite injuries and chronic wounds, many athletes continued sports participation, indicating the potential for injury recurrence and the necessity for proactive treatment. Purification and LC–MS analysis confirmed the molecular weight of the native glycosylase-depolymerase protein. Structural similarities between TP84_26 glycosylase-depolymerase and bacteriophage TP-84 molecules were elucidated through Western blotting. Recombinant TP84_26 glycosylase-depolymerase demonstrated effective destruction of bacterial envelopes, which play a significant role in bacterial virulence.

**Conclusion and References** Comprehensive podiatric assessment is pivotal for managing lower limb injuries in rugby players. Informed decision-making can enhance performance and foot health for athletes despite the challenges posed by recurrent injuries. Additionally, findings from this study offer valuable insights into potential therapeutic applications of TP-84 depolymerase in combating bacterial infections, thereby contributing to advancements in medical and molecular biology.

**References**

1. Łubkowska B., et.al (2023). Microbial Cell Factories, 22(1), 80.
2. Łubkowska, B. et.al (2024). International Journal of Molecular Sciences, 25(2), 722.

## A12 Genetics and Epigenetics (GE): Gene–Gene Interactions of Interleukins and BMI in Physically Active Men

### Maculewicz E.*^[1]^, Garbacz A.^[2]^, Bojarczuk A.^[3]^

#### ^[1]^Military Institute of Aviation Medicine ~ Warsaw ~ Poland, ^[2]^Faculty of Animal Genetics and Conservation, Warsaw University of Life Sciences ~ Warsaw ~ Poland, ^[3]^Faculty of Physical Culture, Gdansk University of Physical Education and Sport ~ Gdansk ~ Poland

##### Correspondence: Maculewicz E.

*Sports Medicine – Open* 2025, ***11(1)***: A12

**Background** In a random Polish adult population sample, 62% of men and 43% of women are found to be overweight [1]. The factors influencing the risk of overweight in physically active individuals are complex and determined by multiple genes.

**Objective/Aim** This study aimed to analyze gene–gene interactions of selected marker genes to evaluate overweight/obesity predisposition in men.

**Methods** We focused on men because the data regarding the male population was particularly concerning. 125 military students lived under standardized conditions, representing a similar level of physical activity and nutrition at the age of 19–25. The participants were divided into groups depending on their BMI (normal vs over BMI > 25). DNA isolation was performed from cheek swabs, and genotyping was carried out for IL1B (rs1143634), IL1RN (rs2234677), IL6 (rs1800795, rs1143627), IL10 (rs1518111, rs3024505, rs3024498) within the genes encoding interleukins. Multivariate dimension reduction (MDR) was used as a statistical method to explore and analyze interactions between Single-Nucleotide Polymorphisms (SNPs) across different chromosomes. With the aid of software such as MDR 3.2.0, it was investigated how combinations of SNPs contribute to BMI level [2–5].

**Results** A significant effect was noted for the interaction of several SNPs, including IL1B (rs1143634) (GG), IL1RN (rs2234677) (TC), IL6 (rs1800795) (CC and GC), IL6 (rs1143627) (AG and AA), IL10 (rs1518111) (CC and CT), IL10 (rs3024505) (AA and AG), and IL10 (rs3024498) (TT and CT).

**Conclusion and References**

Interactions between genes influence the phenotypic outcome and predisposes men to overweight and obesity.

**References**

1. Gajewska, D.; Harton, A. Current Nutritional Status of the Polish Population – Focus on Body Weight Status. J Heal. Inequal 2023, 9 (2), 154–160.
2. Maculewicz, E.; Antkowiak, B.; Antkowiak, O.; Borecka, A.; Mastalerz, A.; Leońska-Duniec, A.; Humińska-Lisowska, K.; Michałowska-Sawczyn, M.; Garbacz, A.; Lorenz, K.; Szarska, E.; Dziuda, Ł.; Cywińska, A.; Cięszczyk, P. The Interactions between Interleukin-1 Family Genes: IL1A, IL1B, IL1RN, and Obesity Parameters. BMC Genomics 2022, 23 (1), 1–12.
3. Maculewicz, E.; Antkowiak, O.; Mastalerz, A.; Borecka, A.; Humińska-Lisowska, K.; Garbacz, A.; Lorenz, K.; Szarska, A.; Michałowska-Sawczyn, M.; Dziuda, M.; Cięszczyk, P. IL-6 Polymorphisms Are Not Related to Obesity Parameters in Physically Active Young Men. Genes (Basel). 2021, 12 (10), 1498.
4. Maculewicz, E.; Mastalerz, A.; Antkowiak, B.; Antkowiak, O.; Garbacz, A.; Szarska, E.; Rusin, P.; Cywińska, A.; Białek, A.; Cięszczyk, P. The Effect of IL10 Gene Polymorphism on Obesity Parameters in Highly Physically Active Young Men. Biol Sport 2022, 39 (4), 1117–1125.
5. Maculewicz, E.; Dzitkowska-Zabielska, M.; Antkowiak, B.; Antkowiak, O.; Mastalerz, A.; Garbacz, A.; Massidda, M.; Bojarczuk, A.; Dziuda, Ł.; Cięszczyk, P. Association of Interleukin Genes IL10 and IL10RB with Parameters of Overweight in Military Students. Genes (Basel). 2022, 13 (2), 291.

## A13 Genetics and epigenetics (GE): The Analysis of the Genetic Profile in Elite Point Fighting Athletes

### Proia P.*^[1]^, Pagliaro A.^[1]^, Alioto A.^[1]^, Messina G.^[2]^, Baldassano S.^[3]^

#### ^[1]^Sport and Exercise Sciences Research Unit, Department of Psychology, Educational Sciences and Human Movement, University of Palermo ~ Palermo ~ Italy, ^[2]^Department of Human Sciences and Promotion of the Quality of Life San Raffaele University Rome (Italy) ~ Roma ~ Italy, ^[3]^Department of Biological Chemical and Pharmaceutical Sciences and Technologies (STEBICEF), University of Palermo, Palermo, 90,128, Italy ~ Palermo ~ Italy

##### Correspondence: Proia P.

*Sports Medicine – Open* 2025, ***11(1)***: A13

**Background** The intersection of genetics and athletic performance has garnered significant attention, with a particular focus on how genetic variations known as Performance-Enhancing Polymorphisms (PEPs) affect critical physical traits essential for athletic success. These genetic markers can inform personalized training programs that optimize athletes' physiological characteristics such as endurance, strength, and recovery rates.

**Objective/Aim** The study aims at the influence of genetic predispositions in PPARα, ACE and CK-MM genes on the performance of Point-Fighting (PF) athletes, where a delicate balance of power and endurance is crucial.

**Methods** A comprehensive genetic analysis was conducted on 24 elite international PF athletes, including members from Italian and Norwegian national teams. Athletes provided saliva samples for DNA extraction, and genetic polymorphisms were identified through Polymerase Chain Reaction (PCR) and enzymatic digestion techniques. The Total Genetic Score (TGS) was calculated to quantify each athlete's genetic predisposition toward power-oriented activities. This genetic profiling was correlated with athletes' performances in the Handgrip Strength Test to evaluate the association between genetic factors and physical capabilities.

**Results** The results showed significant differences in the Handgrip Strength Test between males (54.32 ± 6.39) and females (35.2 ± 4.55) (* p* < 0,5), but no significant difference in Genotype Score (GS) between genders, with a higher average value in females. The correlation between grip strength and GS was stronger in females. Genetic analysis revealed differences in ACE gene polymorphism between males and females, with a predominance of the D allele in both sexes (58.33%). The PPARα gene showed a higher frequency of the C allele, especially in females (77.08%). In the CK-MM gene, the AG heterozygote was the most common variant. These results highlight how genetic factors can differ significantly between sexes and influence athletic performance.

**Conclusion and References** Our findings demonstrated a significant correlation between high TGS and superior performance in PF athletes. Polymorphisms in PPARα and CK-MM were frequently associated with athletes who excelled in power aspects of PF, indicating a genetic predisposition that may be advantageous for the anaerobic demands of the sport. Athletes with optimal genetic backgrounds exhibited enhanced strength and endurance capabilities, essential for success in PF competitions.

## A14 Genetics and Epigenetics (GE): Association Between ACTN3 R577X Polymorphism and Endurance Related Phenotype: A Systematic Review and Meta-Analysis

### Saito M.*^[2]^, Zempo H.^[1]^, De Almeida K.Y.^[2]^, Homma H.^[2]^, Deguchi M.^[2]^, Kozuma A.^[2]^, Kikuchi N.^[2]^

#### ^[1]^Department of Nutrition and Dietetics, Tokyo Seiei College ~ Tokyo ~ Japan, ^[2]^Graduate School of Health and Sport Science, Nippon Sport Science University ~ Tokyo ~ Japan

##### Correspondence: Saito M.

*Sports Medicine – Open* 2025, ***11(1)***: A14

**Background** The R577X polymorphism in the α-actinin-3 gene (ACTN3) is well reported as associated with muscle function characteristics, such as muscle strength (Kikuchi et al. 2015). A previous study reported that Actn 3 KO mice presented higher activity of multiple enzymes in the aerobic metabolic pathway (MacArthur et al. 2008). However, the results of the association between ACTN3 R577X polymorphism and endurance related phenotype have not been consistent in human.

**Objective/Aim** The aim of this systematic review and meta-analysis study was to examined the effect of the ACTN3 R577X polymorphism on endurance related phenotype.

**Methods** Relevant studies published before October 14th, 2023 were identified from the PubMed database using the following keywords and Boolean operators: (“endurance” OR “VO2max” OR “running time” OR “maximum oxygen uptake”) AND (“α-actinin 3” OR “ACTN3 R577X genotype” OR “ACTN3 polymorphism” OR “ACTN3” OR “alpha-actinin 3”). Studies that met the following criteria were included: (1) published in English, (2) included human participants, (3) included healthy participants, (4) provided endurance performance measurements, and (5) analysed the ACTN3 R577X genotype. This review considered endurance related phenotype measurement such as VO2max, VO2peak, running time and Yo-Yo test. All analyses were conducted using R (version 4.1.3) and its “meta” package. The Additive genetic model was assessed using a meta-regression model. Dominant (RR + RX vs XX) and Recessive (RR vs XX + RX) models were analysed using a random effects model.

**Results** A total of 3206 subjects from 15 studies were included in the meta-analysis (VO2max and VO2peak: 11 studies, n = 1504, 15 measurements; Yo-Yo test: 2 studies, n = 171, 2 measurements; Running time: 2 studies, n = 1680, 3 measurements). Although VO2max and VO2peak was not observed in significant association with ACTN3 R577X polymorphism in all subjects and models (Additive: * p* = 0.270, Dominant: * p* = 0.153, Recessive: * p* = 0.659), the results were observed higher for XX genotype compared to RR + RX genotype in 694 athletes and active individuals (* p*  = 0.004). Yo-Yo test and running time were not significantly associated with ACTN3 R577X polymorphism in both all subjects and athletes/active individuals.

**Conclusion and References** This systematic review and meta-analysis observed that the XX genotype in ACTN3 R577X polymorphism presented higher VO2max and VO2peak compared with RR + RX genotype in athletes and active individuals.

**References**

1. Kikuchi N, Yoshida S, Min SK, Lee K, Sakamaki-Sunaga M, Okamoto T, Nakazato K. 2015. The ACTN3 R577X genotype is associated with muscle function in a Japanese population. Appl Physiol Nutr Metab. 40(4): 316–322.
2. MacArthur DG, Seto JT, Chan S, Quinlan KG, Raftery JM, Turner N, Nicholson MD, Kee AJ, Hardeman EC, Gunning PW, et al. 2008. An Actn3 knockout mouse provides mechanistic insights into the association between alpha-actinin-3 deficiency and human athletic performance. Hum Mol Genet. 17(8): 1076–1086.

## A15 Genetics and Epigenetics (GE): Novel Approaches Identifies Key Genetic Loci Contributing to Anterior Cruciate Ligament Rupture Susceptibility

### September A.*^[1]^, Dlamini S.^[1]^, Saunders C.^[2]^, Cieszczyk P.^[3]^, Ficek K.^[4]^, Häger C.K.^[5]^, Stattin E.^[6]^, Nilsson K.G.^[7]^, Eynon N.^[8]^, Tirosh O.^[9]^, Feller J.^[10]^, Bope C.D.^[11]^, Chimusa E.R.^[12]^, Collins M.^[1]^

#### ^[1]^HPALS, Division of Physiological Sciences, Department of Human Biology, University of Cape Town ~ Cape Town ~ South Africa, ^[2]^Division of Emergency Medicine, Department of Family, Community and Emergency Care, University of Cape Town ~ Cape Town ~ South Africa, ^[3]^Faculty of Physical Education, Gdańsk University of Physical Education and Sport, Gdańsk, Poland ~ Gdańsk ~ Poland, ^[4]^Faculty of Physiotherapy, Jerzy Kukuczka Academy of Physical Education in Katowice, Katowice, Poland ~ Katowice ~ Poland, ^[5]^Department of Community Medicine and Rehabilitation, Umeå University ~ Umeå ~ Sweden, ^[6]^Department of Immunology Genetics and Pathology, Science for Life Laboratory, Uppsala University ~ Uppsala ~ Sweden, ^[7]^Department of Surgical and Perioperative Sciences, Umeå University, ~ Umeå ~ Sweden, ^[8]^Australian Regenerative Medicine Institute (ARMI), Faculty of Medicine, Nursing and Health Sciences, Monash University ~ Victoria ~ Australia, ^[9]^School of Health Sciences, Swinburne University of Technology, Melbourne ~ Victoria ~ Australia, ^[10]^OrthoSport Victoria, Epworth Healthcare, Melbourne ~ Vicoria ~ Australia, ^[11]^Department of Mathematics and Computer Science, Faculty of Sciences, University of Kinshasa ~ Kinshasha ~ Congo, the Democratic Republic of the, ^[12]^Department of Applied Sciences, Faculty of Health and Life Sciences, Northumbria University ~ Newcastle-upon-Tyne ~ United Kingdom

##### Correspondence: September A.

*Sports Medicine – Open* 2025, ***11(1)***: A15

**Background** The body of evidence suggests there is a genetic component to anterior cruciate ligament (ACL) injury risk. Several genes have been implicated including, structural, regulatory and inflammatory genes. However, less than 100 genetic loci have been implicated in their injury risk.

**Objective/Aim** To use several approaches to unravel the missing heritability of ACL injuries.

**Methods** A biomedical knowledge graph was developed using both tendon, ligament and cell signalling features. This was combined with the data from a whole genome and whole exome sequencing analyses, towards prioritising a list of candidates genes for ACL injury risk. Bioinformatic analyses with 3D protein modelling was used to identify key network partners. A genetic association study was conducted for participants recruited from Australia, Poland, Sweden, and South Africa. Participants included uninjured controls (CON = 584); ACL ruptures (ACL-R = 768) a sub-group with non-contact mechanism of ACL ruptures (NON = 446). ITGB2 (rs2230528 C/T); COL5A1 (rs12722 C/T) and VEGFA (rs699947 C/A, rs2010963 G/C) was genotyped. Data was stratified by sex. Inferred allele combinations were constructed for network partners. Statistical analyses were conducted using programming environment R. The false discovery rate (FDR) procedure was used to adjust for multiple comparisons.

**Results** Bioinformatic analyses and the 3D protein simulations of ITGB2 illustrated flexibility of the ITGB2 wild type and mutant sequence. Significant distributions were seen or gene combinations. C–C-A-G was under-represented in ACL-R (14%, * p* = 3.0 × 10–4; OR:0.93; 95% CI:0.61–1.41), ACL-NON (14%, * p* = 0.002; OR:0.96; 95% CI:0.61–1.51) compared to CON (19%). C-T-C-G was under-represented in ACL-R (6%, * p* = 0.023; OR:1.08; 95% CI:0.75–1.57) and ACL-NON (4%, * p* = 0.003; OR:0.55; 95% CI:0.286–1.06) compared to CON (7%). Similar results were noted for males or females.

**Conclusion and References** The pathway analysis showed the importance of ITGB2 and its potential in ACL homeostasis as it is involved in a number of cell and extracellular matrix signalling in conjunction with COL5A1 and VEGFA. The gene–gene interactions continuously showcased the potential dominant effect from ITGB2, where if a combination has C allele contribution from ITGB2, a reduced ACL risk was noted while a T allele contribution was more often associated with an increased ACL risk. Altogether we hypothesise that a change in the ITGB2 protein potentially alters the communication between ECM proteins and this disruption between the ECM and cells within ligaments may hereby compromise the biomechanical properties of the knee joint.

In conclusion, we have illustrated that using several approaches we can start exploring a functional hypothesis towards understanding the mechanisms of ACL rupture susceptibility. The structural flexibility for ITGB2 highlights the impact of unravelling our understanding of binding interactions, protein stability and conformation, between partners in biological networks.

## A16 Genetics and Epigenetics (GE): Genetic Profile of Team Sport Athletes

### Stebbings G.*

#### Manchester Metropolitan Institute of Sport, Manchester Metropolitan University ~ Manchester ~ United Kingdom

##### Correspondence: Stebbings G.

*Sports Medicine – Open* 2025, ***11(1)***: A16

**Background** Athletic performance is multifactorial, influenced by anthropometric, physiological and psychological phenotypes, all of which are influenced by genetics.

**Objective/Aim** Genomic studies to date have focused predominantly on sprint/power, or endurance athletes, while the genetic contribution to team sports remains relatively understudied. Success in team sports is determined by the combination of physical, psychological and tactical attributes, according to the rules, pitch/court dimensions, number of players and playing positions, and duration of competition, necessitating sport specific research.

**Methods** Case–control, candidate gene and, more recently, genome-wide association studies, have identified numerous genetic variants associated with achieving elite status in team sports and/or athletic performance phenotypes relevant to team sports.

**Results** Unsurprisingly, ACTN3 is the most widely studied variant in the context of team sports, with evidence demonstrating the R allele and RR genotype are influential for success in power-oriented team sports, including elite football and rugby union. Other variants of interest have included those in genes encoding proteins involved in tissue structure and function (COL5A1), body composition (FTO, ADBR2), and regulation of energy metabolism (AMPD1, PPARA, UCP2), vascular function (NOS3, ACE, AGT), and neurological function (DRD3, COMT, BDNF), for which associations with elite status in team sports and/or athletic performance phenotypes relevant to team sports have been reported. The absence of substantial replication studies, however, makes it difficult to determine if reported observations are robust and indicative of genuine associations. Successful athletic performance in team sports is complex, and while individual athletes within a team often demonstrate similar physiological profiles, physical demands can vary between positions and are influenced by tactical strategies during competition. Indeed, genotype differences in ACTN3, FTO and COL5A1 have been observed between elite rugby union athletes of different playing positions as part of the large, multi-institutional RugbyGene project, which is an example of how such international collaborative research efforts can be effective in establishing substantial databases of genotypic and phenotypic variables to accelerate our understanding of the genetics of team sport athletes for meaningful application in practice.

**Conclusion and References** The integration of genetic insights in talent development, response to training, and injury predisposition has great potential for optimising athlete training strategies in team sports. Application of genetic technologies to supplement, not replace, existing physiological and other non-genetic data, would help to inform more personalised management of team sport athletes by facilitating the prescription of training, nutrition, playing load, mitigation of injury risk and return-to-play protocols to optimise individual, and team, performance potential and player welfare.

## A17 Genetics and Epigenetics (GE): Candidate Gene Polymorphisms and Quadriceps Muscle and Tendon Thickness in Spanish Cross-Country Skiers. LES-5G Study

### Yvert T.*^[1]^, Lim T.^[2]^, Bersezio M.^[3]^, Barceló-Guido O.^[3]^, Benady C.^[2]^, Pérez-Ruiz M.^[1]^, Solik A.^[4]^, Iturriaga T.^[3]^, Santiago C.^[2]^

#### [1]Department of Health and Human Performance, Faculty of Sciences of Sport and Physical Activity-INEF, Universidad Politécnica de Madrid ~ Madrid ~ Spain, [2]Department of Rehabilitation, Faculty of Health Sciences and Sport, Universidad Europea de Madrid ~ Madrid ~ Spain, [3]Department of Sport Sciences, Faculty of Health Sciences and Sport, Universidad Europea de Madrid ~ Madrid ~ Spain, [4]Programa de Tecnificación de Esquí de Montaña, Federación Madrileña de Montañismo ~ Madrid ~ Spain

##### Correspondence: Yvert T.

*Sports Medicine – Open* 2025, ***11(1)***: A17

**Background** Cross-country skiing is a sport with a high demand for muscles and tendons, especially in the lower limb. In the literature, muscle properties such as muscle thickness had previously shown moderate-strong relationships with the magnitude of force production (1); however, no conclusive results are known about the relationship between tendon thickness and muscle strength or power. Ultrasonography has been used as a reliable method to easily assess thickness.

Several genes related to muscle–tendon properties present polymorphisms related to incidence, severity, and recovery of noncontact muscle–tendon injuries (NCMI)(2,3). These can be classified into three categories: muscle–tendon repair and regeneration (e.g., GEFT, MMP3, and IL6), muscle fiber properties (e.g., ACTN3) and matrix composition and maintenance (e.g., COL5A1, DCN, and MMP3); and could also be related to muscle and tendon properties.

**Objective/Aim** For this reason, the aim of this study was to determine possible differences in rectus femoris and vastus intermedius muscle thickness, and quadriceps and patellar tendon thickness between polymorphisms ACTN3 rs1815739, COL5A1 rs12722, IL6 rs1800795, GEFT rs11613457, MMP3 rs679620 and DCN rs516115 in top level Spanish cross-country skiers.

**Methods** 19 Spanish cross-country skiers (6 women) aged 13 to 23 years (mean = 16.5 ± 2.5) were analysed. Rectus femoris and vastus intermedius muscle thickness and quadriceps and patellar tendon thickness were measured using B-mode ultrasonography. Ultrasound images were analysed with ImageJ ver.1.54d software.

The genotypes of the polymorphisms ACTN3 rs1815739, COL5A1 rs12722, IL6 rs1800795, GEFT rs11613457, MMP3 rs679620 and DCN rs516115 were analysed using predesigned TaqManTM Assays.

**Results** For the COL5A1 rs12722 analyses, skiers with TT genotype had significantly lower rectus femoris and vastus intermedius muscle thickness in the nondominant leg than the athletes with CC and TC genotypes (2.08 ± 0.46 cm vs. 2.47 ± 0.21 cm, * p* = 0.028, Cohen’s d = 1.51 (high effect) and 1.91 ± 0.46 cm vs. 2.36 ± 0.28 cm, * p* = 0.031, Cohen’s d = 1.48 (high effect); respectively).

Furthermore, for the DCN rs516115 analyses, the skiers with TT genotype had significantly lower quadriceps tendon thickness in the non-dominant leg than the athletes with CC and TC genotypes (0.47 ± 0.08 cm vs. 0.54 ± 0.04 cm, * p*  = 0.026, Cohen’s d = 1.12 (high effect)).

No significant differences were found for the remaining polymorphisms and the other ultrasonography measures.

**Conclusion and References** Our results suggest that cross-country skiers with genotypes previously associated with a higher incidence and severity of NCMI have a reduced thickness of the rectus femoris and vastus intermedius muscle and quadriceps tendon. This highlights the importance of increasing the knowledge of the role of these polymorphisms in muscle–tendon structure and composition to increase performance and reduce the risk of injury.

**References**

1. PMID: 21142282; 2. PMID: 34270833; 3. PMID: 28856171

## A18 Sports and Exercise Medicine and Health (MH): Translational Insights into Metabolic Flexibility: The Impact of Dietary Polyunsaturated to Saturated Fat Ratios on Fatty Acid Metabolism and Insulin Sensitivity

### Carta G.*^[1]^, Murru E.^[1]^, Manca C.^[1]^, Cabboi M.^[1]^, Trinchese G.^[2]^, Cavaliere G.^[2]^, Solinas R.^[3]^, Montisci R.^[3]^, Mollica M.P.^[2]^, Tocco F.^[3]^, Banni S.^[1]^

#### ^[1]^Department of Biomedical Sciences, University of Cagliari ~ Monserrato ~ Italy, ^[2]^Department of Biology, University of Naples Federico II ~ Napoli ~ Italy, ^[3]^Clinical Cardiology and Sport Medicine, Dept of Medical Science and Public Health, University of Cagliari ~ Monserrato ~ Italy

##### Correspondence: Carta G.

*Sports Medicine – Open* 2025, ***11(1)***: A18

**Background** Dietary fats influence lipid and energy metabolism significantly. Saturated fatty acids (SFA) are particularly implicated in adverse metabolic effects. However, the interplay between dietary fatty acids, including polyunsaturated fatty acids (PUFA), and bioactive lipid derivatives such as endocannabinoids (EC) and N-acylethanolamines (NAE) can profoundly affect metabolic homeostasis. These effects are mediated through mechanisms involving peroxisome proliferator-activated receptors (PPAR-α) and the EC system, which are critical for metabolic flexibility (MF)—the ability to switch energy sources depending on availability and demand (1).

**Objective/Aim** This study aims to translationally evaluate the effects of dietary fat composition, specifically the PUFA/SFA ratio, on MF in both Zucker rats (as a model of diet-induced obesity and insulin resistance) and humans, with a particular focus on body fat and metabolic parameters.

**Methods** Preclinical Study: 24 Zucker rats (obese and lean) were fed diets with low (0.48) and high (3.66) PUFA/SFA ratios for 8 weeks. We analyzed tissue and plasma samples for fatty acids, NAE, and EC levels. Metabolic markers including triglycerides, cholesterol, glucose, and insulin were also quantified.

Human Study: 15 healthy middle-aged adults participated in stepwise exercise tests to assess MF through indicators such as the Metabolic Flexibility Index (MFI) and Peak Energy Substrate Oxidation (PESO). Dietary intakes were recorded over a week.

**Results** Rats: Obese rats on a low PUFA/SFA diet showed improved profiles in plasma metabolic markers and increased muscle levels of N-oleoylethanolamine (OEA), which enhances lipid metabolism by activating PPAR-α. Notably, high palmitic acid (PA) levels were observed in liver of obese rats fed both diets (2), suggesting an enhanced de novo lipogenesis induced by insulin resistance.

Humans: An inverse correlation between the dietary PUFA/SFA ratio and PESO was noted, indicating better MF with higher SFA (especially PA) intake (3).

**Conclusion and References** Lowering dietary PUFA/SFA ratios enhanced glucose and lipid metabolism in Zucker rats primarily through increased OEA production, improving MF. Human data also suggest that a balanced diet rich in PA might promote MF and reduce body fat. These findings underscore the potential of specific dietary fats in modulating metabolic health and highlight the nuanced role of PA in nutritional strategies to combat obesity and enhance MF.

**References**

1. Olson K. A. TIBS, 41, 219–230.
2. Carta G. Nutrients 2023, 15, 4761
3. Murru E. REM-23–0022.R1 2023

**Funding Sources**

Project “Kent’Erbas” REG. (UE) 1305/2013 PSR 2014–2020-19/19.2

PNRR M4.C2.1.1 PRIN 2022 "NUTRICLA" Project N° 2022FWP3XN CUP F53D23007100006.

## A19 Sports and Exercise Medicine and Health (MH): Effects of Acute Normobaric Hypoxia During Exercise and Concurrent Mental Stress on the Cardiovascular System in Healthy Athletes: A Preliminary Study

### Doneddu A.*^[3]^, Ghiani G.M.^[1]^, Pazzona R.^[2]^, Manca A.^[2]^, Pintori S.^[2]^, Ruggiu M.^[4]^, Porceddu M.^[4]^, Fanni M.^[3]^, Guicciardi M.^[2]^, Tocco F.^[4]^, Mulliri G.^[1]^

#### ^[1]^Department of Medical Sciences and Public Health, University of Cagliari (Italy) ~ Cagliari ~ Italy, ^[2]^Department of Education, Psychology and Philosophy, Faculty of Humanities, University of Cagliari (Italy) ~ Cagliari ~ Italy, ^[3]^International PhD in Innovation Sciences and Technologies, Department of Medical Sciences and Public Health, University of Cagliari (Italy) ~ cagliari ~ Italy, ^[4]^Department of Medical Sciences and Public Health, School of Sport Medicine, University of Cagliari (Italy) ~ Cagliari ~ Italy

##### Correspondence: Doneddu A.

*Sports Medicine – Open* 2025, ***11(1)***: A19

**Background** In activities such as hypoxic training, alpinism, or military aviation, the potential combination of hypoxia, exercise, and mental stress could be challenging for the cardiovascular system. However, data are lacking on the effects of this complex scenario on hemodynamic parameters.

**Objective/Aim** This pilot research studied the combined effects of exercise, normobaric hypoxia, and cognitive load on hemodynamic parameters.

**Methods** Six athletes (three female, mean age 29 ± 5.9) underwent our protocol which consisted of a preliminary cardiopulmonary exercise test (CPET) on a cycle ergometer to measure the peak oxygen uptake (VO2peak) and peak workload (Wattpeak), followed by a 20-min exercise session at 40% of their Wattpeak. At the 8th and 18th minute of exercise, a 3-min mental task was administered. All tests were conducted on 2 separate days within a hypoxic tent supplied by two hypoxic generators, simulating sea-level (NORMO) and 3000-m altitude (HYPO) oxygen content. Data on Stroke Volume (SV), Heart Rate (HR), Cardiac Output (CO), Pre-Ejection Period (PEP), Ventricular Ejection Time (VET), Ventricular Ejection Rate (VER), Diastolic Time (DT), Ventricular Filling Rate (VFR) were measured via impedance cardiometry, blood pressure by manual sphygmomanometry, and peripheral blood oxygen saturation (SpO2%) by finger pulse oximetry. Systemic vascular resistance was calculated from CO and mean blood pressure (MBP). Paired sample T-tests assessed differences in VO2peak and Wattpeak. A two-way ANOVA evaluated differences between variables during the various phases of the 20 min-exercise session (factor "time") and between NORMO and HYPO conditions (factor "condition"). Bonferroni’s multiple comparison test compared means individually. A p-value < 0.05 indicated statistical significance.

**Results** On the CPET, Wattmax and VO2peak were higher in NORMO than in HYPO condition as expected (NORMO 275.83 Watt ± 97.18 VS HYPO 247.5 Watt ± 83.89, * p* < 0.05; NORMO 47.32 ml*min^-1^*kg^-1^ ± 5.56 VS HYPO 40.18 ml*min^-1^*kg^-1^ ± 4.86, * p* < 0.05). However, the studied parameters did not significantly vary during the different phases of the 20-min exercise session, with no difference between conditions as well, except for decreased SpO2% during HYPO.

**Conclusion and References** Our results showed no differences in cardiovascular measures while exercise, normobaric hypoxia, and cognitive load act concurrently, indicating good hemodynamic tolerance to this stressful condition in an athletic population. However, the study is currently underpowered, necessitating an increased sample size for definitive conclusions.

**References**

1. Mulliri et al. Acute Exercise with Moderate Hypoxia Reduces Arterial Oxygen Saturation and Cerebral Oxygenation without Affecting Hemodynamics in Physically Active Males. Int J Environ Res Public Health. 2022 Apr 10;19(8):4558.
2. Wang X et al. Cerebral and neural regulation of cardiovascular activity during mental stress. Biomed Eng Online. 2016 Dec 28;15(Suppl 2):160

## A20 Sports and Exercise Medicine and Health (MH): Preliminary Results on the Effects of Concurrent Acute Normobaric Hypoxia, Exercise and Mental Stress on Pulmonary GAS Exchange Kinetics

### Fanni M.*^[1]^, Tocco F.^[2]^, Doneddu A.^[1]^, Ruggiu M.^[2]^, Porceddu M.^[4]^, Pazzona R.^[5]^, Pintori S.^[5]^, Manca A.^[5]^, Guicciardi M.^[4]^, Ghiani G.^[3]^, Mulliri G.^[3]^

#### ^[1]^Department of Medical Sciences and Public Health, University of Cagliari (Italy), International PhD in Innovation Sciences and Technologies, University of Cagliari (Italy) ~ Cagliari ~ Italy, ^[2]^Department of Medical Sciences and Public Health, University of Cagliari (Italy), School of Sport Medicine, University of Cagliari, Cagliari, Italy ~ Cagliari ~ Italy, ^[3]^Department of Medical Sciences and Public Health, University of Cagliari (Italy) ~ Cagliari ~ Italy, ^[4]^ ~ Cagliari ~ Italy, ^[5]^Department of Education, Psychology and Philosophy, Faculty of Humanities, University of Cagliari (Italy) ~ Cagliari ~ Italy

##### Correspondence: Fanni M.

*Sports Medicine – Open * 2025, ***11(1)***: A20

**Background** Hypoxia combined with physical and mental stress is common in many activities such as military aviation, mountain climbing, and exercise training at altitude. These conditions create a complex stressful situation for the cardiorespiratory system. However, the effects of acute hypoxia during exercise with concurrent cognitive load on pulmonary gas exchange kinetics are still unknown.

**Objective/Aim** The present investigation aimed to study oxygen uptake (VO2), carbon dioxide production (VCO2), ventilation (VE), and some derived parameters during exercise with concurrent mental stress in acute normobaric hypoxia and normoxia.

**Methods** Six athletes (three females, mean age 29 ± 5.9) participated in our protocol, which included a preliminary cardiopulmonary exercise test (CPET) on a cycle ergometer to determine peak oxygen uptake (VO2peak) and peak workload (Wattpeak), followed by a 20-min exercise session at 40% of Wattpeak. A 3-min mental task was delivered at the 8th and 18th minutes of exercise. All tests occurred on 2 separate days inside a hypoxic tent supplied by two hypoxic generators, replicating sea-level (NORMO) and 3000-m altitude (HYPO) oxygen levels.

Data on VO2, VCO2, VE, Respiratory Exchange Ratio (RER), Ventilatory Equivalent for Oxygen (VE/VO2), Ventilatory Equivalent for Carbon Dioxide (VE/VCO2), Oxygen Pulse (OP), Tidal Volume (TV), and Breathing Frequency (BR) were collected using a breath-by-breath gas analyzer alongside peripheral blood oxygen saturation (SpO2%) measures via finger pulse oximetry.

Paired sample T-test evaluated variations in VO2peak and Wattpeak. A two-way ANOVA was employed to detect differences between variables during the different phases of the 20 min-exercise session (factor "time") and between NORMO and HYPO conditions (factor "condition"), followed by a Bonferroni’s multiple comparison test when appropriate. We set a p-value < 0.05 to assess statistical significance.

**Results** Wattmax and VO2peak on CPET were higher in NORMO than in HYPO condition as expected (NORMO 275.83 Watt ± 97.18 VS HYPO 247.5 Watt ± 83.89, * p* < 0.05; NORMO 47.32 ml*min^-1^*kg^-1^ ± 5.56 VS HYPO 40.18 ml*min^-1^*kg^-1 ^± 4.86, * p* < 0.05). However, no differences were detected between variables during the 20-min exercise session, with the only exception of SPO2% which was lower during the HYPO test.

**Conclusion and References** Our data suggests that the concurrent exposition to exercise, hypoxia, and mental stress does not impair pulmonary gas exchange in young athletes. Nevertheless, further experiments are ongoing in our lab to confirm these results with a bigger sample size.

**References**

1. Benoit H et al. Oxygen uptake during submaximal incremental and constant work load exercises in hypoxia. Int J Sports Med. 1997 Feb;18(2):101–5.
2. Delistraty DA et al. Use of graded exercise to evaluate physiological hyperreactivity to mental stress. Med Sci Sports Exerc. 1991 Apr;23(4):476–81. Physiology & Nutrition(PN)

## A21 Sports and Exercise Medicine and Health(MH): Evaluation of Coach-Assisted Training Versus Self-Training in Amateur Swimmers

### Giordo M.P.* ^[1]^ Mulliri G.^[2]^, Ghiani G.M.^[2]^, Massidda M.^[2]^, Calò C.M.^[3]^, Caria M.A.^[2]^, Filippo Tocco^[2]^

#### ^[1]^ Sport and Exercise Science Degree Coutses, University of Cagliari, Cagliari, Italy; [2] ^[2]^ Department of Medical Sciences Public Health; University of Cagliari, ~ Cagliari ~ Italy

##### Correspondence: Giordo M.P.

*Sports Medicine – Open* 2025, ***11(1)***: A21

**Background** Over the past half century exercise physiology research produced a vast and well documented literature concerning the quantification of training effects on a series of anthropometric, muscle skeletal, cardiorespiratory and metabolic parameters in young professional elite as well as in non-elite athletes participating in all kinds of sport competitions. In more recent years these studies have been extended to a population of “older” athletes, currently known as master athletes, participating in sport events such as running, swimming, cycling, rowing and in many other sport disciplines.

**Objective/Aim** The aim of the study was to compare the effects of six months of supervised vs. self-administrated training in two groups of male swimmers (Master and Leisure).

**Methods** For the Master group (MS) the training protocols were administered by a certified swimming coach and also entailed 3 days a week of swimming practice which had as a final goal the competition season. The Leasure Group (LS) were instead regular swimmers involved in a recreational workout without any supervision and any specific goal other than keeping themselves physically fit. A series of anthropometric and metabolic parameters were evaluated by means of an incremental treadmill test until exhaustion before (T0) and after the six months of training (T6).

**Results** At T6 both groups showed a significant increase of their test Total Time (TT; MS = 11.9 ± 13.4%; LS = 8.3 ± 15.5%; p > 0.05) and maximum speed reached (Spmax; MS = 6.5 ± 9.3%; LS = 2.1 ± 10.6%; p > 0.05). Maximal aerobic capacity in MS increased by 12.6 ± 14% while in LS 2.5 ± 5.4% (* p* < 0.05). VO2 consumption at anaerobic threshold was increased by 16.8 ± 16.8% for MS and 2.5 ± 5.4% for LS (* p*< 0.05).

**Conclusion and References** Our results show that the coach-assisted training was more effective to evoke significant physiological adjustments in parameters related to aerobic capacity such as VO2max increase and VO2AT.

**References**

Baker AB and Tang YQ. Aging performance for masters records in athletics, swimming, rowing, cycling, triathlon, and weightlifting. Exp Aging Res 2010;
Trappe S. Master Athletes. Int J Sport Nutr Exerc Metab 2001; 11 Suppl:S196–207
17. Storer TW, Dolezal BA, Berenc MN, Timmins JE, Cooper CB. Effect of Supervised, Periodized Exercise Training vs. Self-Directed Training on Lean Body Mass and Other Fitness Variables in Health Club Members. J Strength Cond Res 2014.

## A22 Sports and Exercise Medicine and Health (MH): Cardiopulmonary Exercise Testing in Healthy Adolescents: Analysis of a Possible Relationship Between Metabolic Flexibility and Lifestyle

### Palmas M.*^[1]^, Ruggiu M.[1], Pani C.[1], Melis S.[2–3], Loviselli A. [2–3], Concu A.[2–3], Tocco F. [1–2-3]

#### [1]Department of Medical Sciences and Public Health, School of Sport Medicine, University of Cagliari, Italy, ^[2]^AC Technologies Ltd, Internet of Things Unit, Cagliari, Italy, ^[3]^Sporting Life & Medicine Lab, Sardegna Ricerche, Cagliari, Italy

##### Correspondence: Palmas M.

*Sports Medicine – Open* 2025, ***11(1)***: A22

**Background** Cardiopulmonary exercise testing (CPET) has acquired a major role in evidencing cardiac, pulmonary, and neuromuscular diseases and assessing cardiorespiratory fitness. However, scientific literature still lacks a large and standardized database about healthy children and adolescents1,2.

**Objective/Aim** Our aim was to develop a complex and standardized database that included CPET data, the International Physical Activity Questionnaire score (IPAQ score), body mass index (BMI), fat mass (FM) and fat free mass (FFM) index. Furthermore, metabolic flexibility index (MFI) and Peak Energy Substrates’ Oxidation (PESO) were estimated from cardiopulmonary data3.

**Methods** 45 adolescents aged 16–18 years old (30 females and 15 males) were enrolled for this study. Weights and heights were measured, and every subject’s body composition was assessed using a bioimpedance meter. Cycle ergometer CPET were performed using a 15 W per minute protocol for female subjects and a 20 W per minute protocol for male subjects. Heart rate was continuously measured using a heart rate monitor and blood pressure was assessed every two minutes with a manual sphygmomanometer. Moreover, each subject completed her/his own IPAQ.

**Results** IPAQ score didn’t show any correlation with CPET and body composition data. PESO showed a positive correlation with VO2max (p value ≤ 0,01), VO2max/Kg, VO2max/FFM and respiratory exchange ratio (RER) at peak (p value ≤ 0,001). The MFI didn’t show any correlation with the parameters above, apart from RER at peak (Spearman coefficient -0,4087; p value ≤ 0,01).

**Conclusion and References** In conclusion, our preliminary data address to the use of PESO as a valuable predictor of fitness and metabolic performance. Further investigation on the role of IPAQ and MFI are necessary as concerns this population.

**References**

1. GRIFFITH, Garett J., et al. A Reference Equation for Peak Oxygen Uptake for Pediatric Patients Who Undergo Treadmill Cardiopulmonary Exercise Testing. The American Journal of Cardiology, 2024, 212: 41–47.
2. BURSTEIN, Danielle S., et al. Normative values for cardiopulmonary exercise stress testing using ramp cycle ergometry in children and adolescents. The Journal of Pediatrics, 2021, 229: 61–69. e5.
3. MURRU, Elisabetta, et al. Indirect Calorimetry-Based Novel Approach for Evaluating Metabolic Flexibility and Its Association with Circulating Metabolic Markers in Middle-Aged Subjects. Nutrients, 2024, 16.4: 525.

## A23 Sports and Exercise Medicine and Health (MH): Multiparametric Evaluation of Cardiopulmonary Function at Submaximal Workloads in Pediatric Patients with Congenital Heart Disease

### Perra G.*^[1]^, Zedda F.^[1]^, Arcais F.^[1]^, Sanna P.^[1]^, Concas A.^[1]^, Fiutem J.F.^[2]^, Strainic J.^[2]^, Lai N.^[1]^

#### ^[1]^Department of Mechanical, Chemical and Materials Engineering, University of Cagliari ~ Cagliari ~ Italy, ^[2]^Case Western Reserve University and Rainbow Babies and Children’s Hospital OH ~ Cleveland ~ United States of America

##### Correspondence: Perra G.

*Sports Medicine – Open* 2025, ***11(1)***: A23

**Background** Cardiopulmonary exercise testing (CPET) slopes such as dVO2∕dWR, dVO2∕dHR and dVe/dVCO2 were proposed to evaluate O2 delivery/utilization, cardiac function and ventilatory control using cycle ergometer (1) and treadmill protocols (2). This approach allows for the assessment of cardiorespiratory function with data recorded at submaximal exercise intensities in diseased patients who cannot reach maximal VO2 (VO2,max).

**Objective/Aim** To evaluate cardiopulmonary exercise testing (CPET) slopes: dVO2∕dWR, dVO2∕dHR and dVe/dVCO2 at submaximal workload in pediatric patients (age 13–15 yr) with congenital heart disease: Aortic stenosis, aortic regurgitation (AS-AR), and cardiomyopathies (CMP).

**Methods** AS-AR, CM and healthy patients (C) underwent a CPET with a ramp-type treadmill protocol. The heart rate (HR), oxygen consumption (VO2), carbon dioxide release (VCO2), and ventilation (Ve) were measured whereas the workload (WR) was estimated by a quantitative relationship based on biomechanical relationship that relies on the speed and inclination of the treadmill. The slopes dVO2∕dWR, dVO2∕dHR and dVe/dVCO2 were determined by linear regression of the data recorded until the gas exchange threshold occurred.

**Results** The body mass index was similar in C, AS-AR, and CMP groups (20.5 ± 2 C, AS-AR 20.3 ± 3 and CMP 21 ± 4 kg m-2). AS-AR and CMP patients showed a lower VO2,max (41 ± 8 AS-AR, 36 ± 10 CMP vs. 48 ± 9 C, mL kg-1 min^-1^) in comparison to the control group. The dVO2/dWR (11 ± 2 AS-AR, 8.2 ± 2 CMP vs. 10.5 ± 3 C, mL kg^-1^ Watt^-1^), dVO2/dHR (18.6 ± 6 AS-AR, 16.5 ± 5 CMP vs. 17 ± 6 C, mL beat^-1^) and dVe/dVCO2 (27 ± 4 AS-AR, 27 ± 6 CMP vs. 26 ± 4 C) (* p* <0.001) slopes were similar among the three groups.

**Conclusion and References** The quantitative analysis of the CPET slopes reveals that cardiorespiratory function is not compromised at submaximal workloads in patients with cardiac diseases although maximal aerobic capacity is less than that observed in the control group. CPET slopes are a promising approach to evaluate whether pediatric patients present exercise limitations at submaximal workloads and support decision in prescribing exercise intensity to these patients.

Supported by Sardinia government.

**References**

1. Cooper DM, Leu SY, Galassetti P, and Radom-Aizik S (2014). Dynamic interactions of gas exchange, body mass, and progressive exercise in children. Med. Sci. Sports Exerc. 46:5, 877–886.
2. Lai N, Fiutem J, Pfaff N, Salvedego D, Strainic J (2021). Relating cardiorespiratory responses to work rate during incremental ramp exercise on treadmill in children and adolescents: sex and age differences. European Journal of Applied Physiology 121: 2731–2741.
3. Hansen D, Abreu A, Ambrosetti M, Cornelissen V, Gevaert A, Kemps H and more (2022). Exercise intensity assessment and prescription in cardiovascular rehabilitation and beyond: why and how: a position statement from the Secondary Prevention and Rehabilitation Section of the European Association of Preventive Cardiology. European Journal of Preventive Cardiology, 29:1 230–245.

## A24 Sports and Exercise Medicine and Health (MH): Kartagener’s Syndrome and Aerobic Exercise, is Oxygen Crucial? A Case Report

### Ruggiu M.*^[1]^, Ghiani G.M.^[1]^, Palmas M.^[1]^, Solinas R.^[2]^, Tocco F.^[1]^

#### ^[1]^Department of Medical Sciences and Public Health, School of Sport Medicine, University of Cagliari ~ Cagliari ~ Italy, ^[2]^Clinical Cardiology and Sport Medicine, Department of Medical Science and Public Health, University of Cagliari, 09042 Monserrato, Italy ~ Monserrato (CA) ~ Italy

##### Correspondence: Ruggiu M.

*Sports Medicine – Open* 2025, ***11(1)***: A24

**Background** Primary ciliary dyskinesia is a rare autosomal recessive inherited disease with the congenital abnormality of the primary cilia.

Situs inversus totalis (complete mirror-image reversal of the thoracic and abdominal organs) is present in 50% of patients with primary ciliary dyskinesia.

The triad of situs inversus, bronchiectasis, and chronic sinusitis became known as Kartagener’s syndrome.

**Objective/Aim** In this case study we aimed to report the effects of coach– assisted aerobic exercise training in a patient with Kartagener’s syndrome.

The patient, a 57 years old Caucasian male (1.70 m, 66 kg, BMI 23.5), with stable moderate effort dyspnea, obstructive dysventilation syndrome, and latent respiratory failure, was advised by his pneumologist for training with oxygen therapy. As he was used to practice physical activity without inhaling oxygen reporting subjective well-being, he rejected to use it while training.

**Methods** To assess the effects of his training without oxygen, we carried out two cardiopulmonary tests within eight months. In October 2023 he decided to increase the coach-assisted indoor training: for 3 times a week a 10-min warm-up on the bike followed by exercises on the upper, lower body, and core were performed, then he ended the training with a cool down consisting in 10 min of stretching. Written consent to publish was obtained from the athlete.

**Results** The cardiopulmonary tests (with Bruce’s treadmill protocol) were carried out in May 2023 and January 2024. Before starting each test, basic blood count, body composition assessment, and spirometry were acquired.

In May 2023 values were:

VO2/Kg 21,5 ml/min/kg (62% of the predicted value), Anaerobic threshold (AT) 18,5 ml/min/Kg (58% of the predicted value), the heart rate at AT was 136 bpm, O2 pulse 11 ml/beat, VE/VCO2 slope 25.7. Ventilation 48 L/min (54% of the predicted value). We repeated the cardiopulmonary test after 8 months and these values were detected:

VO2/Kg 24,4 ml/min/kg (76% of the predicted value), Anaerobic threshold (AT) 17,7 ml/min/kg, the heart rate at AT was 121 bpm, O2 pulse 10 ml/beat, VE/VCO2 slope 27.2. Ventilation 62.4 L/min (70% of the predicted value).

**Conclusion and References** Reassuming after 8 months of training the patient increased his VO2/Kg by 12%, the ventilation by 23% and the heart rate at AT decreased by 15 bpm. As these are excellent predictors of improved cardiorespiratory fitness(1), our clinical and instrumental tests and related scientific literature suggest that the patient can continue practicing non-competitive sports without the use of oxygen(2).

**References**

1. Kodama S, Saito K, Tanaka S, et al. Cardiorespiratory fitness as a quantitative predictor of all-cause mortality and cardiovascular events in healthy men and women: a meta-analysis. JAMA. 2009;301(19):2024–2035
2. Cakmak A, Inal-Ince D, Sonbahar-Ulu H, Bozdemir-Ozel C, Tekerlek H, Saglam M, Calik-Kutukcu E, Vardar-Yagli N, Yalcin EE, Ozcelik U, Arikan H. Aerobic exercise training in Kartagener's syndrome: case report. J Exerc Rehabil. 2019 Jun 30;15(3):468–471.

## A25 Sports and Exercise Medicine and Health (MH): Collision Events Across Different Playing Standards and Age Groups in Portuguese Male Rugby Union Players

### Vaz L.*, Vicente João P.

#### University of Trás-os-Montes and Alto Douro and Research Centre in Sports Sciences, Health Sciences and Human Development (CIDESD). ~ Vila Real ~ Portugal.

##### Correspondence: Vaz L.

*Sports Medicine – Open* 2025, ***11(1)***: A25

**Background** Collisions in rugby union have a high injury incidence and burden, and are also associated with player and team performance. Understanding the frequency and intensity of these collisions is therefore important for coaches and practitioners to adequately prepare players for competition.

**Objective/Aim** This study aimed to quantify and compare the collision events across different playing standards and age groups in Portuguese male rugby union players. The successful performance of collision events is fundamental to rugby union and has been associated with team success.

**Methods** Forty rugby union matches, across three male playing standards (i.e., elite, amateur and education), and five age categories (i.e., senior, under [U]18, U16, U14 and U12) were sourced, collected and coded. A one-way analysis of variance was performed to identify differences in considered variables across multiple ages and standards. A post-hoc analysis of least squared difference was used to explore differences among groups, and Cohen’s d was calculated as the effect-size measure.

**Results** The results showed that elite playing standards had a greater number of collisions (e.g., total collisions, tackles, rucks, mauls, and lineouts) events than amateur and education playing standards. The frequency of collision events was greater within higher playing standards (e.g., elite) and older age categories (e.g., senior and U18) compared to lower standard (e.g., education) and younger age categories (e.g., U16 to U12).

**Conclusion and References** These findings provide a comprehensive insight into the characteristics and activity of rugby union match-play and demonstrate that characteristics of the elite senior rugby union differ from younger age categories.

## A26 Physiology & Nutrition (PN): NMR-Based Metabolomics Approach to Study the Metabolic Response to a Session of Mild Dynamic Exercise Under Normobaric Moderate Hypoxia

### Cesare Marincola F.*^[1]^, Libonati V.^[1]^, Masu D.^[1]^, Doneddu A.^[2]^, Fanni M.^[2]^, Ghiani G.^[2]^, Vargiu R.^[3]^, Isola R.^[3]^, Roberto S.^[2]^, Marini E.^[4]^, Rinaldi A.^[4]^, Mulliri G.^[2]^

#### ^[1]^Department of Chemical and Geological Sciences, University of Cagliari ~ Cagliari ~ Italy, ^[2]^Department of Medical Sciences and Public Health, University of Cagliari ~ Cagliari ~ Italy, ^[3]^Department of Biomedical Sciences, University of Cagliari ~ Cagliari ~ Italy, ^[4]^Department of Life and Environmental Sciences, Neuroscience and Anthropology Section, University of Cagliari ~ Cagliari ~ Italy.

##### Correspondence: Cesare Marincola F.

*Sports Medicine – Open* 2025, ***11(1)***: A26

**Background** Hypoxic training (HT) is an altitude training strategy aimed at improving physical performance at sea level. Its widespread adoption among elite athletes is attributed to readily available hypoxic devices facilitating the rapid simulation of high-altitude conditions during sea-level training without the need for acclimatization. However, the efficacy of this method remains a subject of ongoing debate among researchers, lacking a unified consensus. Therefore, further research is needed to fully understand the effects of HT on performance and develop safe and effective training protocols with benefits for athletes.

**Objective/Aim** Recently, we have examined the hemodynamic response of athletes during a session of mild dynamic exercise under moderate normobaric hypoxia (1), showing that the circulatory system was able to handle this hypoxic condition well. In the current study, we extended our previous research by using a metabolomics approach to assess the effects of the above-mentioned exercise performed under moderate normobaric hypoxia on human metabolome.

**Methods** Thirteen physically active individuals (mean age 29.7 ± 4.5 years, mean BMI 23.5 ± 1.4 kg/m2) were recruited to participate in a single session of mild dynamic exercise conducted in normobaric hypoxia (FiO2 = 13.5%), and under normoxia (FiO2 = 21%) for comparison. During both exercise sessions, participants wore a mask connected to a hypoxic gas generator and cycled on an ergometer for 3 min at a workload corresponding to 30% of their Wmax achieved during an incremental cardiopulmonary exercise test (CPT). The exercise session was preceded by a 4-min rest period and followed by 6 min of recovery. Blood and urine samples were collected before the test and within 5 min after concluding the exercise session, and analyzed using 1H nuclear magnetic resonance (NMR) spectroscopy.

**Results** Univariate and multivariate statistical analysis of NMR datasets revealed significant changes in the levels of different metabolites following the hypoxic session. Specifically, a decrease in branched-chain amino acids, phenylalanine, and tyrosine was observed in plasma, alongside an increase in lactate, succinate, citrate, and 3-hydroxybutyrate. Urine analysis indicated an elevation in 3-hydroxyisobutyrate, alanine, lactate, succinate, acetone, and glycine, while dimethylamine and hippurate decreased.

**Conclusion and References** These results are interpreted in relation to changes in metabolic pathways linked to energy metabolism and underscore the potential of NMR metabolomics in enhancing comprehension of the underlying adaptations occurring in response to exercise.

**References**

1. Mulliri G., Magnani S., Roberto S., Ghiani G., Sechi F., Fanni M., Marini E., Stagi S., Lai Y., Rinaldi A., Isola R., Vargiu R., Spranger MD, Crisafulli A. (2022). Acute Exercise with Moderate Hypoxia Reduces Arterial Oxygen Saturation and Cerebral Oxygenation without Affecting Hemodynamics in Physically Active Males. Int. J. Environ. Res. Public Health. 19, 4558.

## A27 Physiology & Nutrition (PN): Experimental Analysis of Nutritional Aspects and Changes in Body Composition During 21 Marathons in 21 Days. A Case Study

### Ghiani G.M.*^[1]^, Mattana D.^[2]^, Ruggiu M.^[3]^, Catta G.^[2]^, Porceddu M.^[3]^, Pazzona R.^[4]^, Tocco F.^[3]^

#### ^[1]^University of Cagliari, Medical Sciences and Public Health Department ~ Cagliari ~ Italy, ^[2]^University of Cagliari ~ Cagliari ~ Italy, ^[3]^Department of Medical Sciences and Public Health, School of Sport Medicine, University of Cagliari ~ Cagliari ~ Italy, ^[4]^Department of Education, Psychology and Philosophy, Faculty of Humanities, University of Cagliari ~ Cagliari ~ Italy.

##### Correspondence: Ghiani G.M.

*Sports Medicine – Open* 2025, ***11(1)***: A27

**Background** Participation in multi day endurance running (MDER) events puts the athlete in front of multiple difficulties. The ability to carry out such events depends also on proper nutrition that aim at maintaining the body composition and energy intake. Literature data often report a caloric deficit among participants in ultra-endurance events with weight and fat mass loss. The conclusion of various studies is that Endurance and Ultra-Endurance athletes are often suffering from a lack of calories and nutrients, as demonstrated by weight and fat mass loss at the end of competitions(1).

**Objective/Aim** Meeting the daily caloric requirement to optimize recovery is the main nutritional challenge of such an event. Our study explores the changes in body composition, basal metabolism, and hydration of an amateur athlete who completed 21 marathons in 21 subsequent days. The goal for the 21 stages was to maintain a constant body composition and to prevent catabolism, in consideration of the high energy expenditure from exercise and that associated with post-exercise, we opted for a slightly high-caloric diet.

**Methods** In August 2022 a young swimmer (age 21) began the training to complete the challenge of running, in July 2023, 21 marathons in 21 days. He asked support to the Sport Physiology Lab of Cagliari University in athletic, nutritional, and psychological, preparation, in order to reach an optimum level of fitness and complete this challenge. We planned his diet, both for the training months and for the MDER event, after assessing his body composition as well as his usual energy intake during the training and the MDER challenge. We detected his body composition, measured by bioimpedance and Plicometry, and we mesured his base metabolic rate before and after the event. Written consent to publish was obtained from the athlete.

**Results** Results found differed from those found in literature(2,3), in particular there was no weight loss while there was an increase in fat mass.

**Conclusion and References** The goal of supporting our athlete during the preparation and realization of an MDER event, preserving his state of health and body composition, can surely be considered as fully achieved. The athlete, despite the considerable pitfall of the challenge, completed it fully respecting the schedule. The constancy in the values of FM and FFM, derived both from plicometry and bioimpedance analysis, before and after the event, was a statement of an adequate energy and macro and micronutrients input. Specifically, we believe that providing an adequate protein intake could have played a fundamental role in counteracting the catabolism associated with exercise and would have favored recovery between one stage and another.

The lipid and glucidic supply guaranteed a physiological response of the endocrine system, while ensuring the functionality of the immune system (4) and avoiding the onset of respiratory diseases, often reported in athletes of Ultra-Endurance races (5).

**References**

1. Kruseman et al. 2005
2. Tiller 2019.
3. Williamson 2016.
4. Carfagno, D. G. & Hendrix, J. C. 2014.
5. Khodaee et al.2012.

## A28 Physiology & Nutrition (PN): Functional, Anthropometric and Nutritional Quality Profile IN Elite Skiers of the Madrid Mountaineering Federation

### Iturriaga T.*^[1]^, Bersezio M.^[1]^, Lim T.^[2]^, Barceló-Guido O.^[1]^, Benady C.^[2]^, Bustamante-Sanchez Á.^[1]^, Solik A.^[3]^, Santiago C.^[2]^, Yvert T.^[4]^

#### ^[1]^Department of Sport Sciences, Faculty of Sport Sciences, Universidad Europea de Madrid, Madrid, Spain. ~ Madrid ~ Spain, ^[2]^Department of Rehabilitation, Faculty of Sport Sciences, Universidad Europea de Madrid, Madrid, Spain. ~ Madrid ~ Spain, ^[3]^Programa de Tecnificación de Esquí de Montaña, Federación Madrileña de Montañismo, Madrid, Spain. ~ Madrid ~ Spain, ^[4]^Department of Health and Human Performance, Faculty of Sciences of Sport and Physical Activity-INEF, Universidad Politécnica de Madrid, Madrid, Spain. ~ Madrid ~ Spain.

##### Correspondence: Iturriaga T.

*Sports Medicine – Open* 2025, ***11(1)***: A28

**Background** The cross-country skiing originated in the tenth century in Norway and Russia, where it was used as a means of travel in harsh winters. Recently it has undergone a profound transformation with the emergence of new technical steps and a new style: the skating style, which together with the classic style constitutes modern cross-country skiing (1). This evolution has increased the scientific interest in this sport, and many studies have been carried out to understand what are the determining factors of performance (2).

**Objective/Aim** The aim of this work was to establish the functional, anthropometric and nutritional profile of young Spanish elite cross-country skiers.

**Methods** Skiers from the Madrid Mountaineering Federation were recruited. Cardiovascular fitness level was evaluated by ergospirometry with an incremental treadmill test on skis (3). To assess upper body isometric strength a handgrip with dynamometer was used. To assess lower body power-strength the Counter Movement Jump (CMJ) test was used with a Chronojump® platform. Body composition was measured with Dual X-Ray Densitometry (DXA) from which the following variables were extracted, Body Adiposity Index (BAI), Fat Mass Index (FMI), Visceral Adipose Tissue (VAT area) and Total Fat (Kg), Skeletal Muscle Mass Index (SMI), Appendicular Skeletal Muscle Index (ASM) and total lean mass (Kg). To determine nutritional quality, a food frequency questionnaire (FFQ) was implemented, where it was categorized by level of compliance in intake of different food groups, as Good (> 75%), Fair (50–75%), and Poor (< 50%). Hydration was classified as Good (> 4 servings) or Poor (3 or fewer servings) based on liquid intake/day.

**Results** A sample of 17 skiers (5 women) were analysed, showing: a mean age of 16.50 ± 2.50 (14–24); weight 61.1 ± 6.7 kg and height 171.9 ± 7.45 cm. The cardiovascular fitness level was 59.8 ± 6.46 ml/kg/min for women and 75.5 ± 6.46 ml/kg/min for men. Upper body strength was 30.7 ± 4.45 kg for women and 39.4 ± 5.91 kg for men; and CMJ test was 23.8 ± 3.36 cm for women and 29.5 ± 6.40 cm for men. Regarding body composition, women skiers presented values of: BMI: 21.5 ± 0.980 kg/m2; fat mass: 15.9 ± 1.37 kg; BAI: 27.4 ± 1.11%; FMI: 5.84 ± 0.416 kg/m2; Lean mass: 39.9 ± 1.15 kg; SMI: 14.7 ± 0.600 kg/m2; ASM: 6.49 ± 0.329 kg/m2; VAT: 35.8 ± 7.91cm2 and men values of: BMI: 20.3 ± 1.56 kg/m2; fat mass: 8.96 ± 2.48 kg; BAI: 14.6 ± 2.99%; FMI: 2.94 ± 0.711 kg/m2; Lean mass: 49.9 ± 6.05 kg; SMI: 16.5 ± 1.25 kg/m2; ASM: 7.66 ± 0.329 kg/m2; VAT: 41 ± 4.71cm2. Finally, 41% of the skiers had a good quality of nutrition, 47% regular and only 5% bad. 82% had a good hydration.

**Conclusion and References** Identifying a functional, anthropometric, and nutritional profile of elite cross-country skiers could serve to understand the determinants of performance in this sport and guide future research and training practices.

**References**

1. PMID: 26,923,666; 2. PMID: 34,649,265; 3. PMID

## A29 Physiology & Nutrition (PN): Impact of Grass-Fed Sheep Cheese on Metabolic Flexibility In Healthy Middle-Aged Individuals. The Kent’erbas Study

### Murru E.*^[1]^, Carta G.^[1]^, Manca C.^[1]^, Cabboi M.^[1]^, Solinas R.^[2]^, Montisci R.^[2]^, Tocco F.^[2]^, Banni S.^[1]^

#### ^[1]^Department of Biomedical Sciences, University of Cagliari ~ Monserrato ~ Italy, ^[2]^Clinical Cardiology and Sport Medicine, Department of Medical Science and Public Health, University of Cagliari ~ Monserrato ~ Italy

##### Correspondence: Murru E.

*Sports Medicine – Open* 2025, ***11(1)***: A29

**Background** Metabolic flexibility (MF) is the ability of the body to optimally use and store different energy substrates according to fluctuations in metabolic demands or energy requirements (1). Dietary fatty acids (FAs) impact MF primarily through their metabolites, which modulate the activity of Peroxisome Proliferator Activated Receptor α (PPARα) and the endocannabinoid (EC) system, influencing both metabolism and food intake. Conjugated linoleic acid (CLA), a distinctive FA found in higher concentrations in dairy and meat from grass-fed ruminants but also synthesized endogenously by human intestinal microbiota, activates PPARα (2) and decreases circulating EC levels. Recent studies have shown that CLA enhances MF in mice by activating PPARα (3).

**Objective/Aim** This study, part of the Kent’Erbas project, aimed to evaluate the effects of cheese from grass-fed sheep on MF in healthy adults aged 45–65. The focus is on the modulation of metabolic parameters.

**Methods** In a double-blind crossover study, 20 participants were divided into two groups. Over 4 weeks, one group consumed 350 g/week of Kent'Erbas cheese, rich in CLA, while the control group consumed a low-CLA cheese from intensive farming systems. After an 8-week washout, the groups switched diets. MF was assessed using a novel method involving stepwise exercise tests on a cycle ergometer connected to a calorimeter (4). Biomarkers for PPARα activation (e.g., N-oleoylethanolamine OEA) and EC system markers (e.g., 2-arachidonoylglycerol 2-AG) were measured in red blood cells via liquid chromatography and tandem mass spectrometry.

**Results** Participants consuming Kent'Erbas cheese showed significantly higher levels of CLA and OEA and lower levels of 2-AG compared to controls. The ratio of OEA to 2-AG, reflecting the balance between PPARα and EC systems, increased by 90%. Both the MF Index (MFI) and Peak Energy Substrate Oxidation (PESO) rates improved by 27% in the Kent'Erbas group.

**Conclusion and References** Four weeks of dietary intervention with Kent'Erbas cheese significantly enhanced MF in middle-aged adults by modulating key metabolic regulators. This suggests that cheese from grass-fed sheep could be strategically used to personalize nutrition and exercise interventions aimed at improving metabolic health.

**References**

1. Goodpaster, B.H. et al., Cell Metab 2017
2. Moya-Camarena, S.Y., et al., J Lipid Res 1999
3. Trinchese, G., et al., Cells 2020.
4. Murru E., et al., Nutrients 2024

**Funding Sources**

Project “Kent’Erbas” REG. (UE) 1305/2013 PSR 2014–2020-19/19.2

PNRR M4.C2.1.1 PRIN 2022 "NUTRICLA" Project N° 2022FWP3XN CUP F53D23007100006.

## A30 Physiology & Nutrition (PN): Body Composition, Body Symmetry, and Body Image Satisfaction in Long-Term Resistance Training Practitioners

### Stagi S.*^[1]^, Campa F.^[2]^, Garau A.^[1]^, Deidda C.^[1]^, Succa V.^[1]^

#### ^[1]^University of Cagliari ~ Cagliari ~ Italy, ^[2]^University of Padova ~ Padova ~ Italy

##### Correspondence: Stagi S.

*Sports Medicine – Open* 2025, ***11(1)***: A30

**Background** In recent years, attention to the physical well-being of athletes has focused not only on the physical and performance aspects, but also on the psychological aspects related to external social pressures, including those specific to the sport context (Burgon et al., 2023).

**Objective/Aim** The aim of this study was to analyse a sample of regular resistance training (RT) exercisers in terms of physical (body composition and body symmetry) and psychological (body image perception and satisfaction) aspects.

**Methods** The sample included 30 RT subjects of both sexes (16 men, age: 31.4 ± 4.3 years; 14 women, age: 30.5 ± 6.1 years) who had been performing resistance training for a long time (men 7.8 and women 6.3 years on average). Anthropometric measurements (height, body mass, arm, waist, and calf circumferences) and bioelectrical measurements (resistance and reactance) of the whole body and segments (arms and legs) were recorded. Body composition was analysed using specific bioelectrical impedance vector analysis (specific BIVA; Buffa et al., 2013). Body image was assessed using a silhouette scale (Williamson et al., 2000).

**Results** Compared to the reference population of Italian and Spanish young adults (Ibáñez et al., 2015), the sample of RT participants had lower fat mass (shorter vector lengths) and higher muscle mass (higher phase angle). Sex differences were found in phase angle (* p* < 0.001, indicative of intracellular/extracellular water ratio or muscle mass) but not in vector length (* p* = 0.206, indicative of fat mass percentage). Segmental body composition (arms, legs) of the right and left side showed no differences in all comparisons. The RT sample showed low body image satisfaction (only 14% of women and 25% of men satisfied), with different patterns between the sexes. Specifically, women want to lose weight (ideal: smaller silhouettes) and men want to gain muscle (ideal: larger silhouettes).

**Conclusion and References** This study shows that the long-term practice of resistance training appears to positively affect body composition by reducing fat mass and improving muscle mass gain and body symmetry. In terms of body image the effect of physical activity does not seem to be influential in terms of improvement in body image satisfaction.

**References**

1. Buffa R. et al. Accuracy of specific BIVA for the assessment of body composition in the United States population. PLoS One. 2013;8(3):e58533.
2. Burgon H. et al. Body image concerns across different sports and sporting levels: A systematic review and meta-analysis. Body Image. 2023;46:9–31.
3. Ibáñez M.E. et al. New specific bioelectrical impedance vector reference values for assessing body composition in the Italian-Spanish young adult population. Am J Hum Biol. 2015;27(6):871–6.
4. Williamson D.A. et al. Body image assessment for obesity (BIA-O): development of a new procedure. Int J Obes Relat Metab Disord. 2000;24(10):1326–32.

## A31 Training and Testing (TT): A Mechatronic Tool For Laboratory Assessing Maximum Exercise Aerobic and Anaerobic Power Out in Élite Cyclists

### Manuello Bertetto A.*^[1]^, Rosadelli S.^[1]^, Roux A.^[1]^, Tocco F.^[2]^, Fois A.^[3]^, Dell'Osa A.^[4]^, Melis L.^[5]^, Marcello R.^[5]^, Concu A.^[5]^

#### ^[1]^Politecnico di Torino ~ Torino ~ Italy, ^[2]^Università degli Studi di Cagliari ~ Cagliari ~ Italy, ^[3]^Nomadyca Ltd ~ Kampala ~ Uganda, ^[4]^Universidad Nacional de Tierra del Fuego ~ Ushuaia ~ Argentina, ^[5]^AC Technologies Ltd ~ Cagliari ~ Italy

##### Correspondence: Manuello Bertetto A.

*Sports Medicine – Open* 2025, ***11(1)***: A31

**Background** In the context of cycling sports, one of the goals to be achieved is to measure the power developed on the bike during training or racing. In the 80 s Ulrich Schoberer developed the “SRM spider” device which, when applied to the bicycle, allows the development of power during a cycling performance to be acquired with very high precision.

**Objective/Aim** To acquire new knowledge on the biomechanical profile of élite cyclists subjected to exhaustive efforts in laboratory race simulations trougth a dedicated mechatronic toot. This was achieved from tests evaluating the progressive changes in power out and its distribution that make up the motion and the oxygen consumption, all remotely acquired by an ICT platform.

**Methods** *Subjects.* Ten skilled cyclists aged between 18 and 27, 69.9 ± 7.1 mean body mass and skilled in road racing, were engaged. *Instrumentation.* The Elite Direto XR-T power meter (Elite Ltd, Italy) was chosen which substitutes bicycle’s rear wheel directly connecting the bike to its cassette. Working mode with constant resistant torque was chosen by cyclists regulating their cadence and gear ratios. This mechatronic tool allows communication with smartphones via the ANT + connection (ANT-Productions, Belgium), from which it traced: speed reached, pedaling cadence and power delivered. Bike of each athlete was used to avoid incorrect pedaling due to an incorrectly sized frame. Acquired data were saved on a Garmin cycle computer and read on the Strava program. The mechatronic tool software calculated the critical power (CP) or anaerobic threshold, double prime (W') representing anaerobic reserve and theoretical maximum oxygen consumption (VO2max). *Experimental protocol.* Athletes performed the following operations: warm-up 20 min on the bike; calibration of instruments; 5 min pedaling constantly at maximum sustainable power (P5’); 30 min unloading and cool-down (power < 100W); 12 min pedaling constantly at maximum sustainable power (P12’); end of the test.

**Results** Data acquired showed a M ± SD of P5' = 352 ± 41 W; P20' = 296 ± 37 W; CP = 262 ± 3.5 W; W' = 24,120 ± 4838 J; VO2max = 60.2 ± 5.5 ml/min/kg; CP/kg = 3.78 ± 0.46 W/kg; W'/kg = 357 ± 73 J/kg.

**Conclusion and References** Our athletes average CP + SD (4.23 W/kg) is practically the same as that found in a recent peer reviewed study from Andereson et al.1 (4.52 W/kg) in 6 cyclists performing a similar indoor protocol and same cyclists had an anaerobic capacity of about 299 J/kg or only 16% lower than in our ones. Moreover, in élite road cyclists performing a pursuit cycling race2 VO2max was about 65 ml/kg/min or only 7% higher than in our athletes. It can be concluded that our mechatronic tool could be reasonably utilized to test in laboratory the physical performance in élite cyclists.

**References**

1. Anderson PE et al. Eur J Appl Physiol. 122:263–265. 2022.
2. Dal Monte A and Faina M. Valutazione dell’Atleta. UTET, Torino-Italy. 1999.

## A32 Training and Testing (TT): Neuromuscular and Neurocognitive Performance in Youth Volleyball Players Across Different Playing Positions

### Mura S.*^[1]^, Tocco F.^[2]^, Massidda M.^[1]^

#### ^[1]^Sport and Exercise Sciences Degree Courses, University of Cagliari ~ Cagliari ~ Italy, ^[2]^Department of Medical Science and Public Health, University of Cagliari ~ Cagliari ~ Italy

##### Correspondence: Mura S.

*Sports Medicine – Open* 2025, ***11(1)***: A32

**Background** Volleyball is an intermittent team ball sport that places emphasis on explosive movements [1,2] in a relatively dynamic and changing environment, which suggests the involvement of high perceptual–cognitive demands during a volleyball match [3]. Consequently, the integration of speed actions and cognitive components are essential in volleyball because allow the ordering, positioning and decision making when carrying out specific actions such as, for example, creation of spaces and blockages. Volleyball players are specialized for their specific position on the field, based on anthropometric characteristics, skills qualities, and physical abilities [4]. Several studies analyzed the anthropometric characteristics and the somatotype of youth female volleyball players but the neuromuscular and neurocognitive factors in relation to playing position have not been fully explored.

**Objective/Aim** The purpose of this study was to investigate the anthropometric, neuromuscular and neurocognitive characteristics of young female volleyball athletes according to playing position. Considering that volleyball players are specialized for their specific position on the field, we hypothesized that differences exist in anthropometric, neuromuscular and neurocognitive characteristics according to playing position of the athletes.

**Methods** A cross-sectional design was followed to collect the data. A total of 57 female youth volleyball players (15.1 ± 1.1 years) from “Serie D” of the Italian Volleyball Federation participated in the study. Anthropometric variables such as weight and stature were measured following the guidelines of the International Society for the Advancement in Kinanthropometry (ISAK). Neuromuscular characteristics were assessed, including hand grip strength (HGS), 5 m sprint, and long jump (LJ). Furthermore, the reactive neurocognitive performance of the lower extremity was measured using the Hold That Squat Test (HTST) in the Blaze Pod light system (BlazePodTM Technology).

**Results** Significant differences (* p*< 0.05) were found among the 5 positional roles. The results indicated that the middle blockers and opposite hitters were the heaviest players, whereas the libero players were the lightest. Moreover, the middle blockers were also the tallest players while the liberos were the shortest. Differences were also found in 5 m’ sprint test, with liberos significantly fastest than setters (* p* = 0.021) and middle blockers (* p*  = 0.010). Finally, middle blockers performed a greater number of hits in the HTST than opposite hitters (* p* = 0.039). No significant differences (p > 0.05) were found among groups for the strength and other reactive neurocognitive performance parameters (Reaction Time and Missing Hits).

**Conclusion and References** Our results demonstrate that significant differences exist in anthropometric, speed and neurocognitive characteristics among playing positions in youth female sub-elite volleyball players. From a practical point of view, it is important that sport scientists and coaches take neuromuscular, neurocognitive and anthropometric characteristics of youth volleyball players into account when designing individualized position-specific training programs.

**References**

1. Marques, MC, González-Badillo, JJ, and Kluka, D. In-season strength training male professional volleyball athletes. Strength Cond J 28 (6): 6–12, 2006.
2. Marques, MC, van den Tillaar, R, Vescovi, JD, and González-Badillo, JJ. Changes in strength and power performance in elite senior female professional volleyball players during the in-season: A case study. J Strength Cond Res 20: 563–571, 2008.Barnes et al. 2007;
3. Nuri L., Shadmehr A., Ghotbi N., Attarbashi Moghadam B. Reaction Time and Anticipatory Skill of Athletes in Open and Closed Skill-Dominated Sport. Eur. J. Sport Sci. 2013;13:431–436. 10.1080/17461391.2012.738712.
4. Paz GA, Gabbett TJ, Maia MF, Santana H, Miranda H, Lima V. Physical performance and positional differences among young female volleyball players. J Sports Med Phys Fitness. 2017 Oct;57(10):1282–1289. 10.23736/S0022-4707.16.06471-9. Epub 2016 Jul 6. PMID: 27,385,546.

## A33 Training and Testing (TT): Movement Demands in Elite Male and Female Beach Volleyball: Sex Differences Positional Analyses on Portugal Championship

### Vincente João P. * Vaz L., Mota M.P.

#### Sport Science Department, Exercise and Health, University of Trás-os-Montes and Alto Douro / Research Center of Sports Science, Health and Human Development (CIDESD), Vila Real – Portugal

##### Correspondence: Vincente João P.

*Sports Medicine – Open* 2025, ***11(1)***: A33

**Objective/Aim** The aim of this study was to quantify the physical demands of beach volleyball (BV).

competition, with reference to the specific sex differences in performance, during elite.

championship match play.

**Methods** 160 professional female n = 80 and Male n = 80 BV players were equipped with a commercially available 10 Hz GPS device containing an inertial measurement unit (Minimax S4, Catapult Sports, Australia). Data collection occurred over 52 official national championship matches, during a single competition, with a total of 120 sets used in the match average analysis. Distance covered (m), meterage per minute (m.min^−1^), Player Load (A.U), and both moderate (20 – 40 cm) and high-intensity Jump volume (> 40 cm) Meterage min (m) and Max velocity (m.min^−1^) were included in the analysis.

**Results** The results revealed positional differences in physical loading between Males and Females. Males and Females covered differences average distances (940.7 ± 424.3 m and 806.3 ± 318.3 m respectively, * p* > 0.05), however males did so at a greater relative intensity, p > 0.05 (36.6 ± 4.1 and 26.9 ± 2.7 m.min^−1^, males and females respectively). Player Load values were differences between sex, with male having only slightly higher values than females (123.3 ± 56.1 and 102.8 ± 44.1 A.U. respectively * p* < 0.05). However, Males analysis revealed high-intensity jump volumes (26.6 ± 13.0 and 12.0 ± 6.5 respectively* p*< 0.05) when compared with females (16.5 ± 11.9 and 6.1 ± 3.3 moderate respectively,* p* < 0.05). According Meterage min males revealed greater value (36.8 ± 4.08 and 31.2 ± 3.68 m.min^−1^ respectively,* P* < 0.05) and the last variable, Max velocity, a moderate values (12.5 ± 1.95 and 11.7 ± 1.67 m/s respectively, * p* < 0.05).

**Conclusion and References** Although the movement demands appear similar between sex differences, in elite female and Male BV, there appears to be a greater locomotive intensity demand for the males almost variables. In addition, there appears to be a significantly greater vertical loading component for males to which has implications for different approaches to physical preparation and recovery. Further analysis is necessary to better understand the physical demands associated with sex player role, however the current data may assist coaches in developing individualized programs that better meet the overall and positional specific demands in female and Male Beach Volleyball.

**References**

1. João, Paulo Vicente, Alexandre Medeiros, Henrique Ortigão, Mike Lee, and Maria Paula Mota. 2021. Global Position Analysis during Official Elite Female Beach Volleyball Competition: A Pilot Study; Applied Sciences 11, no. 20: 9382. 10.3390/app11209382

## A34 Training and Testing (TT): Cardiopulmonary Function Assessment in Pediatric Tetralogy of Fallot Patients Using Treadmill Submaximal Exercise Data

### Zedda F.^[1]^, Perra G.^[1]^, Concas A.^[1]^, Fiutem J.F.^[2]^, Strainic J.^[2]^, Lai N.*.^*[1]*^

#### ^[1]^Department of Mechanical, Chemical and Materials Engineering, University of Cagliari ~ Cagliari ~ Italy, ^[2]^Case Western Reserve University and Rainbow Babies and Children’s Hospital OH ~ Cleveland ~ United States of America

##### Correspondence: Lai N.

*Sports Medicine – Open* 2025, ***11(1)***: A34

**Background** Pulmonary oxygen uptake (VO2) adjustment to work rate (WR) and heart rate (HR) are analyzed by CPET slopes such as dVO2∕dWR and dVO2∕dHR for O2 delivery/utilization and cardiac function, respectively. These CPET slopes can be obtained from data recorded at submaximal exercise intensities with ramp-type protocols using either a cycle ergometer (1) or treadmill (2). The CPET slopes represent an alternative to the maximal VO2 (VO2,max) measurement in assessing cardiorespiratory function in pediatric populations with cardiovascular diseases for those subjects who cannot reliably reach VO2,max.

**Objective/Aim** To evaluate cardiopulmonary exercise testing (CPET) slopes: dVO2∕dWR and dVO2∕dHR at submaximal workloads in pediatric Tetralogy of Fallot (TOF) patients in the 14–18 years old age group.

**Methods** TOF and healthy patients (C) underwent a CPET with a ramp-type treadmill protocol. The temporal profile of heart rate (HR) and oxygen consumption (VO2) were measured, whereas the workload (WR) was estimated by a quantitative relationship based on a biomechanical relationship that relies on the speed and inclination of the treadmill. The slopes dVO2∕dWR and dVO2∕dHR were determined by linear regression of the data recorded until the gas exchange threshold occurred.

**Results** The body mass index was similar in both TOF and C groups (20.5 ± 2 TOF vs 20.5 ± 3 C kg m-2). TOF patients showed a lower VO2,max (36 ± 8 TOF vs. 50 ± 8 C, mL kg^-1^ min^-1^) and gas exchange threshold (25 ± 4 TOF vs. 35 ± 6 C, mL kg^-1^ min^-1^) than that observed in the control group * p* < 0.001. The dVO2/dWR (8.5 ± 2 TOF vs. 10.5 ± 2 C, mL kg^-1^ Watt^-1^) slope was lower in TOF patients compared to the control group. Furthermore, TOF patients dVO2/dHR slope (15.0 ± 5 TOF vs. 19 ± 7 C, mL beat^-1^, * p* < 0.001) was significantly lower than C subjects.

**Conclusion and References** The quantitative analysis of the CPET slopes allows the evaluation of cardiorespiratory dysfunction in TOF patients with compromised cardiac function. CPET slopes are a promising alternative to evaluate cardiac dysfunction and alteration of O2 delivery in TOF patients using submaximal and safer workloads.

**References**

1. Cooper DM, Leu SY, Galassetti P, and Radom-Aizik S (2014). Dynamic interactions of gas exchange, body mass, and progressive exercise in children. Med. Sci. Sports Exerc. 46:5, 877–886.
2. Lai N, Fiutem J, Pfaff N, Salvedego D, Strainic J (2021). Relating cardiorespiratory responses to work rate during incremental ramp exercise on treadmill in children and adolescents: sex and age differences. European Journal of Applied Physiology 121: 2731–2741.

Supported by Sardinia government.

## A35 Biomechanics (B): Relationship Between Lower Body Muscular Performance and Body Composition in Youth Soccer Players

### Arippa F.*^[1]^, Pau M.^[1]^, Tocco F.^[1]^, Cugia P.^[2]^, Calò C.M.^[1]^, Massidda M.^[1]^

#### ^[1]^University of Cagliari ~ Cagliari ~ Italy, ^[2]^FIMSI CR Sardegna and Cagliari Calcio Spa ~ Cagliari ~ Italy

##### Correspondence: Arippa F.

*Sports Medicine – Open* 2025, ***11(1)***: A35

**Background** Youth football academies play a pivotal role in identifying future talents in professional sports. Continuous monitoring of young athletes features is essential for optimizing development, guiding tailored training interventions, and enhancing their performance potential. Combined with physical, technical and tactical skills, anthropometrics and body composition features are essential factors considered in the selection process and contributing to in-game success. In soccer, where explosive power and agility of lower body are paramount, indicators of performance such as vertical jumps (VJ) and sprints are also monitored. VJ is indirect indicator of strength, explosive power, and muscle fiber composition in lower limb, while sprint performance provides insights into speed, power, and agility, and are known to correlate with performance in soccer matches. Understanding the interrelationship between anthropometric characteristics, body composition and physical performance in young athletes is crucial for optimizing training protocols and talent identification strategies.

**Objective/Aim** To explore the correlation between lower body muscular performance indicators– evaluated through instrumented versions of countermovement jump (CMJ), squat jump (SJ) and sprints– vs body composition and anthropometrical parameters commonly assessed in soccer youth teams.

**Methods** Forty young soccer players (8–12 years old) were recruited and their anthropometric and body composition features (i.e., stature, body mass, skinfold thickness, arm circumference, body fat percentage BF%, and fat-free mass, FFM) collected. Instrumented sprint test performance across distances ranging from 5 to 30 m was evaluated using photocells. SJ and CMJ were also tested using a wearable inertial sensor. Correlations between anthropometrical and body composition features vs performance tests were explored.

**Results** Moderate correlations between VJ abilities and sprint, increasing for longer distances, were found (r = -0.372 to r = -0.663). VJ performance increased with age, both for the SJ (r = 0.506) and CMJ (r = 0.500), while negative correlations were observed with skinfold thickness across all body areas (ranging from r = -0.360 to r = -0.472) and BF% (SJ r = -0.458, CMJ r = -0.457). Sprint time was negatively correlated with age only for longer distances (20 m r = -0.415, 30 m r = -0.435), and positively correlated with skinfold thickness and BF%, with correlations strengthening as distance increases (r = 0.344 to r = 0.524).

**Conclusion and References** Age and low body fat are related to better performances. While higher body fat percentages appear to have little impact on short sprints, they are determinant for longer distances. Surprisingly, weight did not show any significant correlation, indicating that body composition, rather than total body mass, plays a more critical role in performance tests. Moderate correlations between sprint and VJ performances highlights the necessity of evaluating both when aiming to enhance young players abilities.

## A36 Biomechanics (B): The Role of Balance and Lower Limb Strength in elite Soccer: An Assessment Across Playing Positions and Professional Levels

### Arippa F.*, Porta M., Casu G., Leban B., Pau M.

#### University of Cagliari ~ Cagliari ~ Italy

##### Correspondence: Arippa F.

*Sports Medicine – Open* 2025, ***11(1)***: A36

**Background** Soccer is a multifaceted sport where, in addition to technical skills, strength, coordination and balance play a pivotal role in defining the overall performance, also serving as a benchmark for injury recovery and talent identification. Literature reported the importance of neuromuscular control as key factor able to influence performance and predict the occurrence of lower limb injuries. In particular, the instrumental assessment of postural control effectiveness has been demonstrated useful to discriminate soccer players according to their technical level and expertise, but also valuable marker of fatigue and ankle instability.

Among assessment methods, postural sway and Dynamic Postural Stability Index (DPSI) are used to evaluate static and dynamic balance control, while vertical jumps—Squat Jump (SJ) and Countermovement Jump (CMJ)—represent a consolidated tool to assess athlete physical performance in terms of lower limbs’ explosive strength. Quantitatively examining these skills across various playing positions and competitive levels can highlight their relationship with enhanced performances, helping in training customization, reducing injury risk, and maximizing the team performance.

**Objective/Aim** To explore differences among élite soccer players across various playing positions and professional levels in static and dynamic postural control, assessed through sway parameters and the DPSI, alongside variations in physical performance measured by SJ and CMJ.

**Methods** Static and dynamic balance, as well as physical performance, were evaluated on 116 élite soccer players from professional and youth leagues. The assessments included calculation of sway during static posturography (bipedal and unipedal stances), DPSI following a submaximal forward jump, and instrumented vertical jumps—SJ and CMJ. Performance differences were investigated on derived parameters among players at different levels and roles.

**Results** No differences were observed in static balance across player levels or position. In contrast, DPSI showed significant differences for player level at the dominant limb (* p*< 0.05). Team-level differences were also detected for SJ and CMJ (* p* < 0.001 in both cases). Interestingly, the latter was the only parameter where playing position significantly affected the results (*p* < 0.01), with goalkeepers and forwards having better outcomes than midfielders and defenders.

**Conclusion and References** Dynamic assessment of balance abilities may be more relevant than static tests in understanding the performances of élite soccer players. While static posturography showed no differences across players and levels, dynamic balance, especially on the dominant limb, was influenced by team level. Physical performance, varied between professional leagues and player roles, emphasizing the specificity of these skills in discriminating athletes’ levels. Findings underscore the importance of integrating dynamic tests for a detailed characterization of elite soccer players.

## A37 Biomechanics (B): Bioelectromagnetic Multiphysics Modeling of Tecar Therapy

### Lodi M.B.*

#### University of Cagliari ~ Cagliari ~ Italy

##### Correspondence: Lodi M.B.

*Sports Medicine – Open* 2025, ***11(1)***: A38

**Background**

The Capacitive and Resistive Electric Transfer (TECAR) therapy uses electromagnetic (EM) energy at radiofrequency (RF) regime (300 kHz^-1^ MHz) to generate endogenous heat to treat musculoskeletal disorders in physiotherapy, acting as analgesic, decontracting, and elasticizing therapy [1]. TECAR can be resistive or capacitive, depending on the EM coupling with the human body [2]. The biological effects of the temperature rise (37 °C < T < 39 °C), in the range of mild hyperthermia, result in enhanced skin and muscle, improved blood perfusion and oxyhaemoglobin concentration [3]. Recently TECAR fields of applications have been identified as musculoskeletal physiotherapy, dermato-functional physiotherapy and sports [3]. However, despite more than hundreds of work can be retrieved on the topic, the nature of existing articles does not allow a quantitative analysis. So far, few authors dealt with the theoretical or numerical modeling of TECAR systems and the biological response [4].

**Objective/Aim**

The aim of this work is to fill this knowledge gap and develop a numerical multiphysics framework to quantitatively investigate the TECAR therapy and provide a bioelectromagnetic engineering rationale to study, interpret and advance a physical thermal therapy that is of high relevance to sport science and practice.

**Methods**

A numerical FEM model to solve the Poisson's equation and couple to the Bio-Heat transfer equation to compute the spatio-temporal patterns of the specific absorption rate (SAR) and temperature was developed. The model was validated using data from [1]-[4]. The model can reproduce resistive, capacitive or bipolar electrodes, working at different frequencies. We focused on the investigation of the effects of a moving electrode, as done in clinical practice.

## Results

We found that the simulated peak temperatures, heating rates (± 0.5 °C) agree with the theoretical data from [1], and the experimental findings from [2]-[4]. The electrode speed scarcely affects the SAR and heating patterns.

**Conclusion and References**

The proposed theoretical framework could serve as the basis for empowering the design of innovative and more effective medical devices to carry out this procedure. The numerical findings suggest to carry out studies observing professional operators and acquiring kinematic parameters to refine the simulations and provide useful feedback to physiotherapists.

**Funding**

Matteo Bruno Lodi received funding from the European Union—NextGenerationEU through the Italian Ministry of University and Research under PNRR—M4C2-I1.3 Project PE_00000019 "HEALITALIA" to CUP F53C22000750006 University of Cagliari.

**References**

1. G. Barassi, et al., Integrative Clinical Research, pp. 39–46, 2022. 10.1007/5584_2021_692
2. J. Rodríguez-Sanz, et al., Sci Rep 10.1: 22,290, 2020. 10.1038/s41598-020-78612-8
3. L. De Sousa-De Sousa, et al., Int J Env Res Pub Health 18.23: 12,446, 2021. 10.3390/ijerph182312446
4. J. Jiménez-Lozano, et al., Phy Med & Biol 57.22: 7555, 2012. 10.1088/0031-9155/57/22/7555

## A38 Outdoor Training & Active Sports Tourism (OTAST): Diagnostic Tool For Bone Fracture Risk in Mountain Tourism

### Dell'Osa A.H.*^[1]^, Concu A.^[2]^, Flaherty E.^[3]^

#### ^[1]^Laboratorio de Electrónica Aplicada y Biomedicina—Universidad Nacional de Tierra del Fuego ~ Ushuaia ~ Argentina, ^[2]^AC Technology SRL ~ Cagliari ~ Italy, ^[3]^Hospital Regional de Ushuaia. Ministerio de Salud de la Provincia de Tierra del Fuego, Antártida e Islas del Atlántico Sur ~ Ushuaia ~ Argentina

##### Correspondence: Dell'Osa A.H.

*Sports Medicine – Open* 2025, ***11(1)***: A38

**Background** The city of Ushuaia (Tierra del Fuego, Argentina) presents a geography that mixes mountain, forest and sea. In this scenario, trekking and climbing are extreme sports practiced all year round, while skiing (alpine and cross-country) and snowboarding are only practiced during the winter season. These activities cause each year around 1000 accidents. Studies carried out in the area of Aragon (Spain) (Soteras et al., 2014) show that less than 50% of mountain traumas in the wrist and ankle are bone fractures that prevent the injured person from being able to mobilize. Mobilizing a rescue has a very high logistical cost, which, in the case of the city of Ushuaia is borne by the provincial government or by the volunteers themselves.

**Objective/Aim** The aim of this work is to analyze the possibility of using a portable, low-cost, innocuous and in-situ tool to detect bone fractures in the ankle and/or wrist region using bioimpedance spectroscopic measurements or, as it is technically known, electrical impedance spectroscopy (EIS) in a non-invasive way.

**Methods** This study was carried out at the Hospital Regional de Ushuaia. Measurements were performed on a control group of subjects with both healthy ankles (GH) and on a group of subjects with a diagnosed bone fracture (GF) in one of their ankles. Each group consisted of 18 subjects (22 females and 14 males), the mean age was 37.9 ± 12.1. Only closed, non-exposed and non-comminuted fractures of one ankle were considered while the controlateral ankle was healthy. EIS meter was a home-made bioimpedance meter called GuanacoOA (Dell’Osa, 2022), which measures bipolar bioimpedance between 5 and 100 kHz with a 1 kHz step by ECG disposable electrodes around the ankles area on both extremities, and visualization of measurements has been carried out from a Smartphone with Android 6.0 operating system. Data analysis was approached by Cole model fitting (Grimnes et al., 2014) defined by four parameters: Rs, Rp, Q and α and the Rs was chosen as of clinical importance.

**Results** Mean ± SD of chosen parameters were for GF were: healthy side Rs = 348.71 ± 68.37, fracture side Rs = 238.20 ± 49.02. For GH were: right side Rs = 295.50 ± 47.88, left side Rs = 291.06 ± 45.56. Data shows a significant Rs decrease in fractured ankle regarding the healthy one with * P* = 5.2669·10–6 while in GH there was a P NS (0.3455). From previous results criteria for establish fracture presence is |ΔRs|= 60Ω between fractured and healthy subject. So 15 GF subject 15 a |ΔRs|> 60Ω while only 1 GHgave |ΔRs|> 60Ω.

**Conclusion and References** These results found suggest to be a starting point that confirm the validity of GuanacoOA in leg fractures of mountain tourists as a non-invasive, innocuous, and rapid method. In addition, this device can be operated by non-medical personnel and outside of a health institution since X-ray equipment is not used.

**References**

1. Dell’Osa A. GuanacoOA. OSHWA Certification UIDAR001.2022
2. Soteras I et al. Injury. 46(4):585–589.2014
3. Grimnes S et al. Bioimpedance and bioelectricity basics.2014

## A39 Outdoor Training & Active Sports Tourism (OTAST): Inverse Relationship Among Left Ventricle Stroke Volume and Head Linear Acceleration Induced in Coastal Sailing Tourists By Moored Boat Rolling: A Non-Drug Anti-Hypertensive Advantage?

### Tocco F.^[1]^, Palmas M.^[1]^, Ruggiu M.^[1]^, Solinas R.^[1]^, Manuello Bertetto A.^[2]^, Fois A.*^[3]^, Melis L.^[3]^, Dell'Osa A.^[4]^, Marcello R.^[5]^, Melis S.^[5]^, Concu A.^[5]^

#### ^[1]^Università degli Studi di Cagliari ~ Cagliari ~ Italy, ^[2]^Politecnico di Torino ~ Torino ~ Italy, ^[3]^Nomadyca Ltd ~ Kampala ~ Uganda, ^[4]^Universidad Nacional de Tierra del Fuego ~ Ushuaia ~ Argentina, ^[5]^AC Technologies Ltd ~ Cagliari ~ Italy

##### Correspondence: Fois A.

*Sports Medicine – Open* 2025, ***11(1)***: A39

**Background** It has been found that nervous fibres from the vestibular nuclear neurons (VNN) go towards the nucleus tractus solitarius (NTS) which plays a pivotal role in modulating muscle sympathetic nerve activity (MSNA) in the lower limbs. Those stimuli act in maintaining arterial blood pressure since controlling smooth muscle tone of vessels, thereby modulating the peripheral vascular resistance (PVR) and in turn ventricle afterload regulating. It has been found that MSNA decreases when linear acceleration was sinusoidally applied along head’s naso-ocipital direction coinciding with the space X axis (± Gx) at 0.1 Hz in subjects seated on a sliding chair where MSNA decreases with respect to the peak of applied Gxb.

**Objective/Aim** As moored sailboats are subjected to sinusoidal-like oscillations for the sea wave motion and people seated inside it undergo slow naso-ocipital oscillations, 3 aged sailors staying in a moored sailboat were tested to find possible vestibular induced cardiodynamic chandes.

**Methods** a) Subjects—3 male sailing tourists ageing of 75.0 ± 5.6 years embarked on a coastal cruise lasting 7 days and sleeping on the moored boat. b) Instrumentation—A 3 devices tool was used integrating together an electrical impedance meter to non-invasively assess sailors left ventricle stroke volume (LSV) beat-to-beat; an accelerometer for linear accelerations along the subjects naso-ocipital axis (± Gxn-o); a biosignals ICT platform to remotely acquire all biological signals of any interest.

**Results** An average of 1958 ± 470 size samples of the LSV/ ± Gxn-o couples from all sailors were harvested while sleeping in the sailboat moored and the linear regression method of LSV versus ± Gxn-o was applied to each sailor reaching the following equations: (1) [LSV (ml) = 61.32 – 6.04 Gxn-o (m s-2)]; (2) [LSV (ml) = 64.45 – 14.19 Gxn-o (m s-2)]; (3) [LSV (ml) = 70.36 – 13.29 Gxn-o (m s-2)]. These equations clearly highlight the inverse relationship of LSV versus ± Gxn-o which were statistically significant [* p* = 0.004 in (1), * P* < 0.0001 in (2) and (3)].

**Conclusion and References** In all three subjects tested, for each unit deviation of the value of ± Gxn-o, i.e. 1 m s-2 or about one tenth of the acceleration of terrestrial G, LSV value deviates linearly but inversely by about 11.2 ± 4.6 ml on average. Since it has been found that Gxn-o increase induces a MSNA reduction together with PVR fallc, this could lead to the reduction in LSV. Considering that LSV is the main responsible for the mean arterial blood pressure changes, it could reasonably be speculated that a non-invasive antihypertensive effect might be reached by long lasting stay into a rolling moored boat.

**References**

1. James C et al. Auton Neurosci. 158(1–2):127–131. 2010.
2. Concu A. Research Signpost, Transworld Research Network, Kerala-India, pp. 61–83. 2007.
3. Cui J et al. Am J Physiol Regul Integr Comp Physiol. 281(2):R625-R634. 2001.

## A40 Outdoor Training & Active Sports Tourism (OTAST): Rescues in Adventure Tourism: New Methodology for Continuous Monitoring of Vital Signs

### Villanueva Jousset T.*^[1]^, Concu A.^[2]^, Dell'Osa A.^[1]^

#### ^[1]^Laboratorio de Electrónica Aplicada y Biomedicina. Universidad Nacional de Tierra del Fuego, Antártida e Islas del Atlántico Sur ~ Ushuaia ~ Argentina, ^[2]^AC Technology SRL ~ Cagliari ~ Italy

#### Correspondence: Villanueva Jousset T.

*Sports Medicine – Open* 2025, ***11(1)***: A40

**Background** Mountaineering is defined as "those physical activities that consist of progressing, either ascending or not, through mountainous terrain and are consciously carried out with the purpose of maintaining or improving our health (physical or mental), relating to other people, escaping from everyday life, experiencing sensations driven by the practice itself, or finally, for a desire for self-improvement and/or competition".

Mountaineering is beneficial for health, but can pose risks in hostile, difficult-to-access areas, potentially leading to severe accidents or fatalities.

Medical attention in these situations is limited, as rescue teams may lack of medical doctors during the initial assessment of the injured. Monitoring vital signs such as heart rate, respiratory rate, temperature, and oxygen levels is done with multiparameter monitors that are not ergonomic outside of the hospital setting and require cables for patients that can increase the risk in mountain rescue situations.

**Objective/Aim** Implementing a new methodology for monitoring vital signs in a portable, ergonomic, and easy-to-apply manner for a patient who needs to be transported on a stretcher carried by rescuers.

**Methods** A mask-shaped support has been developed, with electronic sensors capable of measuring saturimetry in the earlobe, temperature non-invasively, respiratory rate, and heart rate. Recorded data are transmitted wirelessly to a smartphone app, which processes and presents it in an accessible way. The App allows the vital data of the rescued person to be sent to the health centre responsible for the definitive patients' care, enabling advanced planning before their arrival.

**Results** In a controlled lab environment, it was observed that the measurements of the parameters obtained by the device are accurately recorded while in motion. This allows the monitored individual to be wrapped up and secured on a stretcher.

In an app reporting of physiological data allows freedom in the use of both hands by the rescuer and, in case of an alarm, records the event and enables on-site decision-making. Measurements were validated with calibrated instruments and vital sign simulators.

**Conclusion and References** This proposal establishes the groundwork for an innovative solution in mountain rescues: a mask equipped with electronic sensors monitoring the vital signs of an injured person, aiming to increase their survival through better monitoring and early reporting to the diagnosing doctors and subsequent treatment. Such technologies fall under the concept of IoT applied to healthcare, leveraging the connectivity provided by current cellular networks.

**References**

1. Moscoso D. La montaña y el hombre en los albores del siglo XXI. Zaragoza. Barrabés Editorial; 2002. 1ª edición.
2. Soteras, I. (2021). Medicalizar los equipos de rescate en montaña: justificación socio-económica en base a la evolución de la mortalidad en el Pirineo Central. Archivos de Medicina del Deporte.
3. Chan, A. (2016). Biomedical Device Technology: Principles and Design. Charles C Thomas Pub Ltd; 2nd edition.

## A41 Psychology, Social Sciences & Humanities (SH): Physical Activity Levels and Quality of Life in Adolescent Students: Novelties From IPAQ AND SF-36 Questionnaire

### Pani C.*^[1]^, Palmas M.^[1]^, Ruggiu M.^[1]^, Melis S.^[2–3]^, Loviselli A.^[2–3]^, Concu A.^[2–3]^, Tocco F.^[1–2−3]^.

#### [1]Department of Medical Sciences and Public Health, School of Sport Medicine, University of Cagliari, Italy, [2]AC Technologies Ltd, Internet of Things Unit, Cagliari, Italy, [3]Sporting Life & Medicine Lab, Sardegna Ricerche, Cagliari, Italy

##### Correspondence: Pani C.

*Sports Medicine – Open* 2025, ***11(1)***: A23

**Background** The World Health Organization (WHO) defines physical activity as any bodily movement produced by skeletal muscles that requires energy expenditure. It is known that physical activity brings numerous benefits to general well-being and improves the quality of life. The IPAQ questionnaire appears to be a valid instrument for measuring physical activity levels. To our knowledge only one research revealed few and weak correspondences between physical activity levels and mental health in adolescents.

**Objective/Aim** Considering the lack of conclusive results comparing physical activity levels and quality of life factors in adolescent people, we propose to correlate physical activity levels and bio-psycho-social well-being, as is measured by the SF 36 questionnaire, in a pool of adolescent students.

**Methods** The research sample included 33 healthy volunteers (26 F-7 M) aged 16 -17 years, attending high school with a biomedical program. The whole group of participants completed the IPAQ and SF 36 questionnaires independently to ensure the reliability of the tests. In particular, the first questionnaire gives us an indication of the level of physical activity or inactivity of the participants, whereas the second questionnaire investigates the following aspects: physical functioning, physical limitations, emotional limitations, energy, emotional well-being, social functioning, pain and general health.

**Results** Analysis of the IPAQ questionnaire, found that 5 individuals are poorly active, 13 normally active and 15 active or very active. 2-way ANOVA statistic was applied to compare the three IPAQ groups with the items of the SF-36 questionnaire. Comparing poorly active with normally active individuals, we observed significant differences in the following aspects: physical limitations (* P* < 0.01), emotional limitations (* P*< 0.05) and energy (* P*< 0.05). Furthermore, when comparing the poorly active participants with the very active ones, we observed significant differences in the following aspects: physical limitations (*P* < 0.05) and emotional limitations (*P* < 0.001).

**Conclusion and References** According to our data, it can be observed that: (1) active or very active adolescents have fewer limitations due to physical and emotional problems than poorly active peers; (2) normally active adolescents have fewer limitations due to physical and emotional problems and have more energy than poorly active peers.

**References**

1. Bull FC, Al-Ansari SS, Biddle S, et al. World Health Organization 2020 guidelines on physical activity and sedentary behaviour. Br J Sports Med. 2020 Dec;54(24):1451–1462.
2. Lee PH, Macfarlane DJ, Lam TH, Stewart SM. Validity of the International Physical Activity Questionnaire Short Form (IPAQ-SF): a systematic review. Int J Behav Nutr Phys Act. 2011 Oct 21;8:115.
3. Biddle SJ, Asare M. Physical activity and mental health in children and adolescents: a review of reviews. Br J Sports Med. 2011 Sep;45(11):886–95.

## A42 Psychology,Social Sciences & Humanities (SH): Exploring the Impact of Ramadan Fasting on Decision-Making and Performance in Elite Athlete Referees: A Case Study

### Pazzona R.*^[3]^, Ruggiu M.^[1]^, Ghiani G.M.^[2]^, Mulliri G.^[2]^, Doneddu A.^[2]^, Massidda M.^[3]^, Pintori S.^[2]^, Porceddu M.^[1]^, Manca A.^[2]^

#### ^[1]^Department of Medical Sciences and Public Health, School of Sport Medicine, University of Cagliari, Italy ~ Cagliari ~ Italy, ^[2]^Department of Medical Sciences, Sports Physiology Lab., University of Cagliari, Italy ~ Cagliari ~ Italy, ^[3]^Department of Medical Sciences and Public Health, University of Cagliari,Italy ~ Cagliari ~ Italy

##### Correspondence: Pazzona R.

*Sports Medicine – Open* 2025, ***11(1)***: A23

**Background** Studies demonstrate significant negative effects of Ramadan fasting on physical performance, mood, body composition, and dietary habits. Despite evidence suggesting potential cognitive impairments due to changes in meal timing, nutrient intake, dehydration, research on the effects of Ramadan fasting on cognitive function in athletes is lacking. Although studies in this area are limited and sometimes inconclusive, some suggest that athletes maintaining their usual routines during Ramadan may not experience significant performance declines.

**Objective/Aim** Analyze the effects on a referee during Ramadan to understand fasting's impact on decision-making among elite athletes. We hypothesized that during Ramadan, the athlete might experience worsened stress, mood, and decision-making, reduced body composition and macronutrient intake, with no significant changes in cardiopulmonary function compared to pre-Ramadan.

**Methods** This case study involved an elite male referee, aged 36. The protocol consisted was administered twice, once before Ramadan and once during Ramadan (29th day). Initially, a preliminary analysis was conducted (anthropometric measurements, ECG, blood pressure, stress-recovery balance). Subsequently, the referee underwent a standard cardio-pulmonary exercise test (CPET) on a treadmill to assess physical capacity. Respiratory parameters were measured using a gas analyzer, and dedicated software was used to evaluate peak oxygen uptake and the first ventilatory threshold. The decision-making test was conducted immediately after reaching the first ventilatory threshold, involving the vocalization of refereeing decisions in video-administered game situations. The CPET ended at exhaustion, followed by Borg scale and mood assessment. Written consent to publish was obtained from the athlete.

**Results** The results show a decrease in fatigue, a worsening of mood in anger, tension and depression and a worsening in decision making. The CPET results were similar pre and during-Ramadan (peak velocity; velocity at VT1; V’O2peak/BM; V’O2peak/FFM; V’O2/BM at VT1; V’O2peak/FFM. The athlete lost one kg of body mass and impedance analysis showed that it came mainly from the loss of extra cellular water, fat mass recorded a slight decrease, while his muscle mass increased.

**Conclusion and References** This study sheds light on the impact of Ramadan fasting on elite male referee, an area previously unexplored. The results indicate significant effects on their psychological, physical, and metabolic well-being. While fatigue decreases, mood and decision-making abilities decline during Ramadan, affecting psychological health. Although physical performance remains steady, changes in body composition underscore metabolic effects. Further research with larger samples is needed to understand these mechanisms and mitigate negative impacts on referees' performance and well-being.

**References**

1. Abaïdia et al., 2020
2. Burke et al., 2012
3. Cherif et al., 2016
4. Chaoucachi et al., 2012
5. Rad et al., 2023
6. Shepard et al., 2013

